# Functional Impacts of *NRXN1* Knockdown on Neurodevelopment in Stem Cell Models

**DOI:** 10.1371/journal.pone.0059685

**Published:** 2013-03-25

**Authors:** Liyun Zeng, Peilin Zhang, Lingling Shi, Vicky Yamamoto, Wange Lu, Kai Wang

**Affiliations:** 1 Zilhka Neurogenetic Institute, Keck School of Medicine, University of Southern California, Los Angeles, California, United States of America; 2 Eli and Edythe Broad Center for Regenerative Medicine and Stem Cell Research, Keck School of Medicine, University of Southern California, Los Angeles, California, United States of America; 3 Department of Biochemistry and Molecular Biology, Keck School of Medicine, University of Southern California, Los Angeles, California, United States of America; 4 Department of Psychiatry and Department of Preventive Medicine, Keck School of Medicine, University of Southern California, Los Angeles, California, United States of America; Johns Hopkins University, United States of America

## Abstract

Exonic deletions in *NRXN1* have been associated with several neurodevelopmental disorders, including autism, schizophrenia and developmental delay. However, the molecular mechanism by which *NRXN1* deletions impact neurodevelopment remains unclear. Here we used human induced pluripotent stem cells (hiPSCs) and human embryonic stem cells (hESCs) as models to investigate the functional impacts of *NRXN1* knockdown. We first generated hiPSCs from skin fibroblasts and differentiated them into neural stem cells (NSCs). We reduced *NRXN1* expression in NSCs via a controlled shRNAmir-based knockdown system during differentiation, and monitored the transcriptome alteration by RNA-Seq and quantitative PCR at several time points. Interestingly, half reduction of *NRXN1* expression resulted in changes of expression levels for the cell adhesion pathway (20 genes, P = 2.8×10^−6^) and neuron differentiation pathway (13 genes, P = 2.1×10^−4^), implicating that single-gene perturbation can impact biological networks important for neurodevelopment. Furthermore, astrocyte marker GFAP was significantly reduced in a time dependent manner that correlated with *NRXN1* reduction. This observation was reproduced in both hiPSCs and hESCs. In summary, based on *in vitro* models, *NRXN1* deletions impact several biological processes during neurodevelopment, including synaptic adhesion and neuron differentiation. Our study highlights the utility of stem cell models in understanding the functional roles of copy number variations (CNVs) in conferring susceptibility to neurodevelopmental diseases.

## Introduction

Recent human genetic studies have demonstrated that copy number variations (CNVs) are associated with several neurodevelopmental and neuropsychiatric disorders [1]–[3]. These CNVs include large-scale, recurrent genomic deletions caused by non-allelic homologous recombination, such as those targeting 1q21.1 [4]–[6], 16p11.2 [7], [8], 15q13.3 [4], [5] and 22q21.2 [9], [10], as well as CNVs impacting single genes, such as exonic deletions in SH3 and multiple ankyrin repeat domains protein 2 (*SHANK2*) [11], neurexin 1 (*NRXN1*) [12] and contactin 4 (*CNTN4*) [13], [14]. Among them, *NRXN1*, especially exonic deletions in the *α-NRXN1* isoform, represents one of the most robust associations for autism [12], [14]–[16], schizophrenia [17]–[19] and other developmental disorders [20], [21]. Therefore, *NRXN1* may play an important role in regulating the neurodevelopmental process, and deletions in *NRXN1* may be involved in the molecular pathophysiology of multiple related disorders.

NRXN1 is a presynaptic neuronal adhesion molecule that interacts with postsynaptic neuroligins in excitatory and inhibitory synapses in the brain, and is involved in synapse formation and maintenance [22], [23]. NRXN1 is the upstream regulator of presynaptic-postsynaptic complex, which include neuroligins (*NLGNs*), SHANKs, postsynaptic density protein 95 (PSD-95) and guanylate kinase–associated proteins (GKAPs). Several components of this presynaptic-postsynaptic complex have also been associated with autism and other neurodevelopmental disorders [11], [24]–[26]. Despite these prior genetic studies, it is still unclear how deletions in *NRXN1* (half dose of *NRXN1*) confer susceptibility to multiple related diseases, and what biological processes are compromised due to *NRXN1* haploinsufficiency. These types of questions may be partially answered in animal models by behavioral and molecular studies (for example, mouse with *NLGN3* deletion [27], 15q13 duplication [28] and *SHANK2* deletions [29], [30]); however, besides the difficulty in generating animal models, it is unknown how these models faithfully represent neurodevelopmental process in humans. Therefore, in addition to other model systems, *in vitro* cellular models (such as neurons derived from humans [31]) could perhaps provide complementary and fine-grained insights into the functional roles of CNVs during neurodevelopment.

Human embryonic stem cells (hESCs) are early developing cell types that have the potential to develop into all types of cells *in vitro* [32]. hESCs may serve as model systems to inform us how typical developmental programs are implemented and how the programs can be modified due to specific genetic mutations. However, due to various concerns, there are few available hESCs lines and there is no bank of hESCs that encompasses the genetic diversity of human populations (including patient populations). In 2007, Yamanaka and colleagues discovered that four transcriptional factors were sufficient to reprogram somatic cells into human induced pluripotent stem cells (hiPSCs) [33]. Gene expression assay, epigenetic markers and cell fate determination potential have suggested that these hiPSCs were highly similar to hESCs [34]. By generating hiPSCs from specific subjects, we have the ability to obtain the tissues of interests by *in vitro* differentiation, which would share the identical genetic background as the subjects from whom hiPSCs were derived from. Besides the potential roles in regenerative medicine [35], hiPSCs can also serve as important research tools in terms of modeling complex diseases, including neurodevelopmental and neuropsychiatric diseases [36]–[39]. For example, in recent years, hiPSCs have already been used for studying Parkinson’s disease [40], Rett syndrome [41], schizophrenia [42], fragile X mental retardation syndrome [43], Timothy syndrome [44], and others.

In the current study, we addressed a central hypothesis that if deletions of *NRXN1* influence neurodevelopment *in vivo*, such effects could be manifested in an *in vitro* system based on human stem cell models. We used both hiPSCs and hESCs to re-create *NRXN1* haploinsufficiency, to address the potential concerns that neurons derived from hiPSCs may contain biases due to the introduction of foreign genes/vectors. Our results demonstrated that neural stem cells (NSCs) derived from both hiPSCs and hESCs can be reliable models for studying neurodevelopment, and that these models can be used to study the functional genetic link of *NRXN1* deletions and neurodevelopment, by regulating gene expression levels. Our study also has implications to the study of functional impacts of other single-gene deletions or large-scale CNVs in neurodevelopmental diseases.

## Materials and Methods

### Establishment of hiPSCs

2.0×10^6^ human fetal dermal fibroblasts (HDFf, acquired from ATCC) were transfected with 4 µg CAG.OSKM-puDtk reprogramming transposon and 2 µg pCyL43 transposase plasmid through nucleofection (Amaxa Nucleofector technology). Transfected cells were cultured on in α-MEM supplement with 10% FBS for 2 days. Then medium was switched to hESCs medium (DMEM/F12 supplement with 20% KSR, L-glutamine, non-essential amino acid and 4 ng/ml FGF2). Medium was changed every 2 days. Starting from week 3, ES-like colonies were manually picked up and plated in irradiated mouse embryonic fibroblast (MEF) feeder layer and fed with hESCs medium daily. The MEF was generated and provided by USC Stem Cell Core.

### Culture of hESCs and hiPSCs

The H9 hESCs (passage 35–45, provided by USC Stem Cell Core) [45] and two hiPSCs clones (passage 1–10, generated at the Lu lab from HDFf mentioned above) were cultured in DMEM/F12, 20% knockout serum replacement (Invitrogen), 1% nonessential amino acids, 0.1 mM β-mercaptoethanol, and 8 ng/mL bFGF (Invitrogen) on X-ray inactive mouse embryonic fibroblasts pre-coated with 0.1% gelatin. hESCs and hiPSCs were passaged both mechanically and chemically. For chemical splitting, CTK splitting medium was used, which was composed with 1 mg/mL collagenase IV (Worthington Biochemical Corp.), 0.25% trypsin without EDTA, 20% knockout serum replacement and 1 mM CaCl_2_ in PBS. Cells were routinely passaged every 3–4 days in 6-well plate, and culture medium was changed every day.

### Neural Stem Cells (NSCs) Induction and Neuronal Differentiation

Embryoid Bodies (EBs) were formed first by splitting the hESCs and hiPSCs colonies into appropriate size and seeding on 6 cm ultra low attachment dish (BD Biosciences) with ES culture media without bFGF, and changing medium every two days. On day 5 of EBs formation, we switched to N2 media (DMED/F12 with N2 supplement (Invitrogen) and 1% penicillin/streptomycin) for targeted differentiation of EBs to neurospheres. On day 10, we collected all the neurospheres and seeded them on prepared Matrigel coated culture dish in N2 media with 20 ng/mL bFGF. The neural rosettes were formed on Matrigel plates after 5–10 days culture. We manually dissected the neural rosettes from the Matrigel plate, and gentally digested them with 0.05% trypsin to break the rosettes to smaller pieces and then seeded on poly-ornithine and fibronectin (Sigma) double coated plate in N2/B27 culture media (50% N2 media (DMED/F12 with N2 supplement (Invitrogen) and 1% penicillin/streptomycin), 50% B27 media (DMEM/F12 with B27 supplement (Invitrogen) and 1% penicillin/streptomycin), with 20 ng/mL bFGF). Spontaneous neuronal differentiation was performed in N2/B27 culture medium without bFGF. The culture media were changed every two days for both NSCs culture and neuronal differentiation.

### Lentivirus Production and Infection of NSCs

Human TRIPZ lentivirus inducible shRNAmir (RHS4740 for *NRXN1*, Open Biosystems) plasmids stock was expanded in LB medium with 100 µg/mL *ampicillin*and purified using Plasmid Maxi kit (Qiagen). The non-targeting TRIPZ lentivirus inducible shRNAmir control was made by integrating the non-silencing scrambled shRNAmir sequence at *MluI* and *XhoI* restriction sites on pTRIPZ vector, which does not match any known mammalian genes following the Open Biosystems shRNAmir manual. The TRIPZ lentivirus plasmids were packed by lentivirus packaging vectors pMD2.g and psPAX.2, and transfected into HEK293T cells using Polyethylenimine (Sigma) as transfection reagent. Lentivirus was collected 48–72 hours after transfection by centrifuge at 28,000 rpm for 1.5 hours at 4°C using Beckman Counter Optima L-100 XP ultracentrifuge. The lentivirus particles were resuspended in PBS and store at −80°C. The lentivirus was titered using HEK 293T cells following the Open Biosystems shRNAmir manual. For the infection of NSCs, we first cultured the NSCs in N2/B27 media. When the cells reached confluence, they were trypsinized and collected in 1.5 mL Eppendorf tube with 150 µl media. The lentivirus was then added to the tube and incubated at 37°C for 1 hour, then re-plated on poly-ornithine and fibronectin double coated 6 cm plate with 2.5 mL media. The cells were incubated overnight and changed to the fresh media the next day morning. To induce the shRNAmir expression, 1 µg/µl Doxycycline (Enzo Life Science) was added to the media. For long term shRNAmir expression, Doxycycline was refreshed every two days.

### Quantitative Real-time-PCR

Total RNA was extracted using RNeasy mini kit, in combination of RNAase-free DNAase to remove the potential genomic DNA contamination (Qiagen). The cell lysate was homogenized by passing 5 times through a blunt 27-gauge needle. RNA concentration was quantified by Nanodrop 1000 Spectrophotometer (Thermo Scientific). Reverse transcription was performed with 1.5 µg RNA using ProtoScript M-MuLV First Strand cDNA Synthesis Kit using random primers (New England Biolabs). The quantitative real-time PCR was carried on with gene-specific primers and *iQ* SYBR Green Supermix using Bio-Rad CFX96 system (Bio-Rad). The mRNA starting quantity was determined by the relative standard curve method using Bio-Rad CFX Manager software. The sequences of primers used were as the following:

NRXN1 α-isoform: Forward: 5′- TCAGGCATTGGACACGCTATGGTA -3′; Reverse: 5′- TAGCCCGTTGTGGTAAGAATCCCA -3′;

TUJ1 [46]: Forward: 5′-CCTGGAACCCGGAACCAT -3′; Reverse: 5′-AGGCCTGAAGAGATGTCCAAAG -3′.

GFAP [47]: Forward: 5′ –CCCACTCTGCTTTGACTGAGC-3′; Reverse: 5′-CCTTCTTCGGCCTTAGAGGG-3′.

PAX6 [48]: Forward: 5′-CCAGAAAGGATGCCTCATAAA-3′; Reverse: 5′-TCTGCGCGCCCCTAGTTA-3′.

OCT4: Forward: 5′-GACAGGGGGAGGGGAGGAGCTAGG; Reverse: 5′-CTTCCCTCCAACCAGTTGCCCCAAA.

NANOG: Forward: 5′-TGAACCTCAGCTACAAACAG; Reverse: 5′- TGGTGGTAGGAAGAGTAAAG.

SOX2: Forward: 5′-AGCTACAGCATGATGCAGGA; Reverse: 5′-GGTCATGGAGTTGTACTGCA.

NESTIN: Forward: 5′- AAGAGAACCTGGGAAAGGGAGAGT; Reverse: 5′- TTCCTGAGCCAGTTCTTGGTCCTT.

GAPDH: Forward: 5′-CATGTTCGTCATGGGTGTGAA-3′; Reverse: 5′-AGTGATGGCATGGACTGTGGT-3′.

The expression of genes of interest was normalized to GAPDH in all samples. After normalization, data were transformed as the target mRNA signal relative to untreated control samples, and then plotted on GraphPad Prism 5.0 (GraphPad Software, Inc).

### Immunocytochemistry

Cells were fixed in 2% paraformaldehyde for 15 min, then washed 3 times with PBS, 5 min each, then incubated in blocking buffer with 10% normal goat serum (NGS) in PBS, for 30 min at room temperature. Primary antibody was added at room temperature for 1 h in 5% NGS blocking solution with 0.1% Triton X-100. The following day, cells were washed 3 times with PBS, 5 min each, and then incubated with goat anti-mouse IgG Cy2- or Cy3-conjugated secondary antibody (Jackson ImmunoResearch Labs Inc.) in PBS at room temperature for 1 h. Then the cells were washed 3 times, 5 min each with PBS, then stained with DAPI at 1∶1000 in PBS at room temperature for 5 min. The cells were washed with PBS and images were taken on Zeiss AX10 fluorescence microscope. The primary antibodies were: anti-TRA-1-60 (MAB4360, Millipore), anti-human Nanog-NL493 (R&D Systems), anti-Oct-4 (SC5279, Santa Cruz), anti-SSEA-4 (MC-813-70, DSHB), anti-NESTIN (MAB1259, R&D systems), anti-TUJ-1 (MMS-435P, Covance) and anti-GFAP (556327, BD biosciences).

### Western Blot

Whole cell lysate was prepared from subconfluent cells resuspended in RIPA buffer (50 mM Tris-HCL, PH 7.6; 1% NP-40; 0.5% Sodium deoxycholate; 150 mM NaCl; 0.1% SDS, 0.5 mM EDTA) and protease inhibitor cocktail (Sigma, MO) on ice for 20 min. Proteins were isolated from insoluble cell debris by centrifuge at 14,000 rpm for 15 min at 4°C. The protein concentration was determined by Pierce Bicinchoninic Acid Protein Assay Kit (Thermo Scientific) and BSA protein standard (Sigma, MO). Protein extract was denatured by 1X Laemmli sample buffer plus 10 mM dithiothreitol (DTT) at 37°C for 30 min. Proteins (10–30 µg) were loaded on SDS-PAGE gels and separated by electrophoresis in Tris-Glycine running buffer on Mini-Protean Tetra Cell (Bio-Rad) at 100 V. Proteins were transferred to 0.2 µm PVDF membrane (Millipore, MA) in ice cooled 4°C Towbin transfer buffer/0.015% SDS/20% Methanol on Criterion Blotter with plate electrodes (Bio-Rad) for 1 h at 100 V. Membrane was blocked in 5% fat free milk in 1X Phosphate Buffered Saline with 0.1% Tween-20 (PBST) for 1 h in room temperature, and then probed with anti-NRXN1 (clone N170A/26, NeuroMab, CA) and anti-actin (sc-1615, Santa Cruz, CA) overnight in blocking buffer at 4°C, and with secondary antibodies (goat anti-mouse conjugate to horseradish peroxidase) for 2 h at room temperature. Signal was developed by SuperSignal West Pico chemiluminescent substrate (Thermo Scientific).

### RNA-Seq Analysis

The total RNAs were also subject to RNA-Seq analysis at selected time points during neuronal differentiation. Briefly, total RNAs were isolated from cell lysate by RNeasy mini kit (Qiagen), and their qualities (OD260/280, rRNA ratio, RNA integrity number) were determined by BioAnalyzer 2100 (Agilent). mRNAs were isolated from 5 µg total RNA by polyT capture, and were used for library construction per Illumina’s protocol. The Illumina HiSeq2000 sequencer was used to generate 91 bp paired end reads on RNA samples, and each sample was sequenced with two technical replicates in two lanes. On average, >60 million paired-end reads were obtained for each sample.

We used TopHat [49] version 1.2.0 for aligning the Illumina short reads against the reference human genome (build 37) as well as a reference GTF file constructed from Illumina’s iGenome annotation archive (http://cufflinks.cbcb.umd.edu/igenomes.html, Homo Sapiens NCBI build 37.2). We filtered the GTF file to remove all annotated mitochondrial and ribosomal RNAs. After generating sequence alignments as BAM files [50], we next used Cuffdiff [51] version 1.1.0 to summarize the gene expression values as FPKM measures and to compare cell lines to identify genes with differential expression. The expression fold change, P-values and false discovery rate (FDR) values were taken from Cuffdiff’s outputs. The list of significantly differentially expressed genes, as defined by FDR<0.01, were analyzed by the DAVID web server [52] for enrichment of Gene Ontology categories. Additionally, the results of gene expression (fold change and P-values) were overlaid with known protein-protein interactions [53], [54] in the Cytoscape software [55] for network-based analysis and visualization.

## Results

### Human iPS Cell Generation Using PiggyBac Transposon Reprogramming System

**Figure 1:**
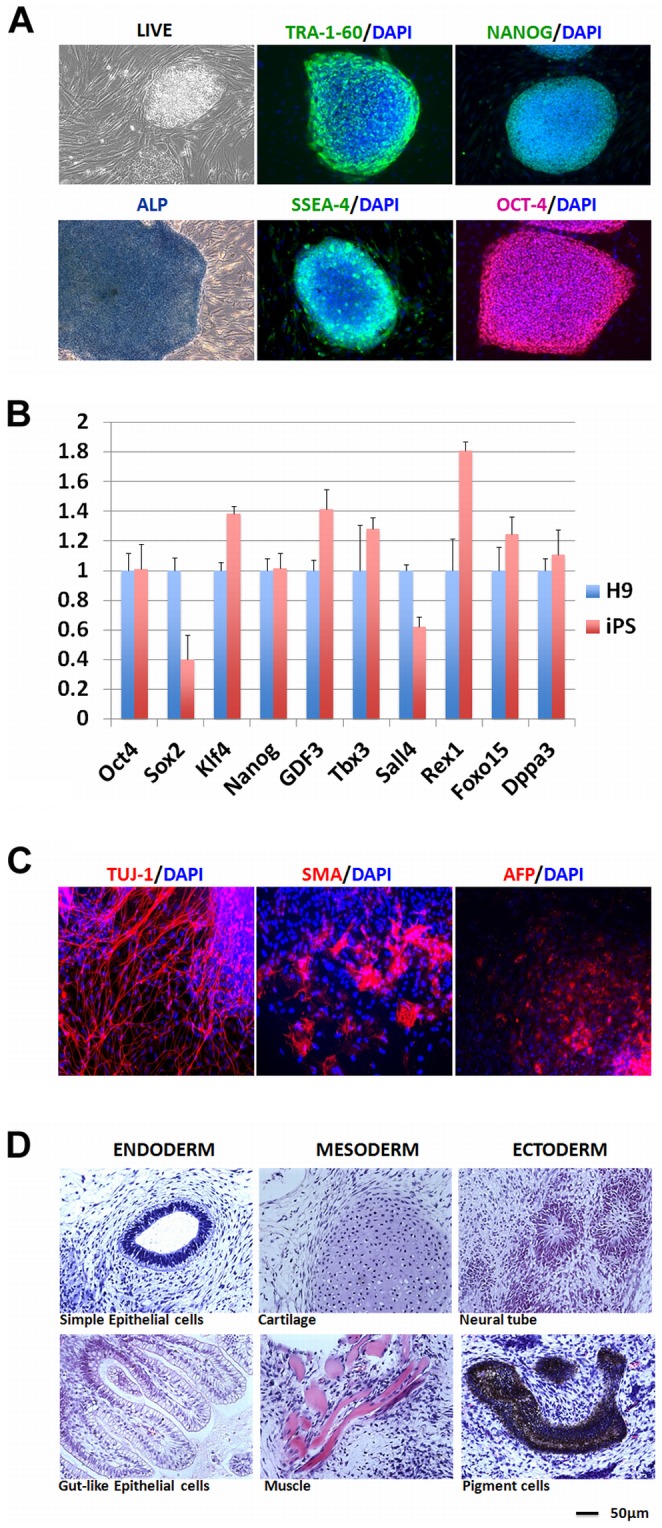
The hiPSCs are fully pluripotent. **A.** immunocytochemistry and alkaline phosphatase (ALP) staining for pluripotent markers. Nuclear markers: *Oct 4* and *Nanog*; Surface markers: *SSEA-4, Tra-1-60*. **B**. qPCR for various pluripotent genes indicates that hiPSCs are very similar to hESCs H9 in terms of gene expression levels. **C.** *in vivo* differentiation of hiPSCs to three germ layers. Ectoderm marker: *TUJ-1*; Mesoderm marker: *SMA*; Endoderm marker: *AFP*. **D.** hiPSCs can form teratoma in mouse containing derivatives of all three embryonic germ layers (ectoderm, mesoderm, and endoderm), shown by histopathology staining.

To establish the iPS model for our study, we used PiggyBac transposon reprogramming system [56] to delivery four reprogramming factors (OCT4, SOX2, KLF4 and c-MYC) into human fetal dermal fibroblast (HDFf) by nucleofection. Human iPS colonies began to emerge in 2 weeks, and we isolated stable hiPSCs manually and characterized them at week 4. The cell behavior of these hiPSCs was almost indistinguishable from hESCs. Immunocytochemistry revealed that these cells expressed all examined pluripotency markers ALP, Tri-1-60, SSEA-4, Oct-4 and Nanog (**Figure 1A**). Quantitative real-time PCR (qPCR) analysis confirmed similar levels of gene expression for pluripotency-related genes in these lines (**Figure 1B**). These cells were fully capable of being differentiated into three germ layers in mouse teratoma *in vivo* (**Figure 1C, D**), and they also maintain the correct chromosome numbers and karyotypes. All these characterization assays demonstrated that we obtained the target hiPSCs successfully.

### Neural Stem Cells Remain their Natural Differentiation Potential and Pattern

**Figure 2:**
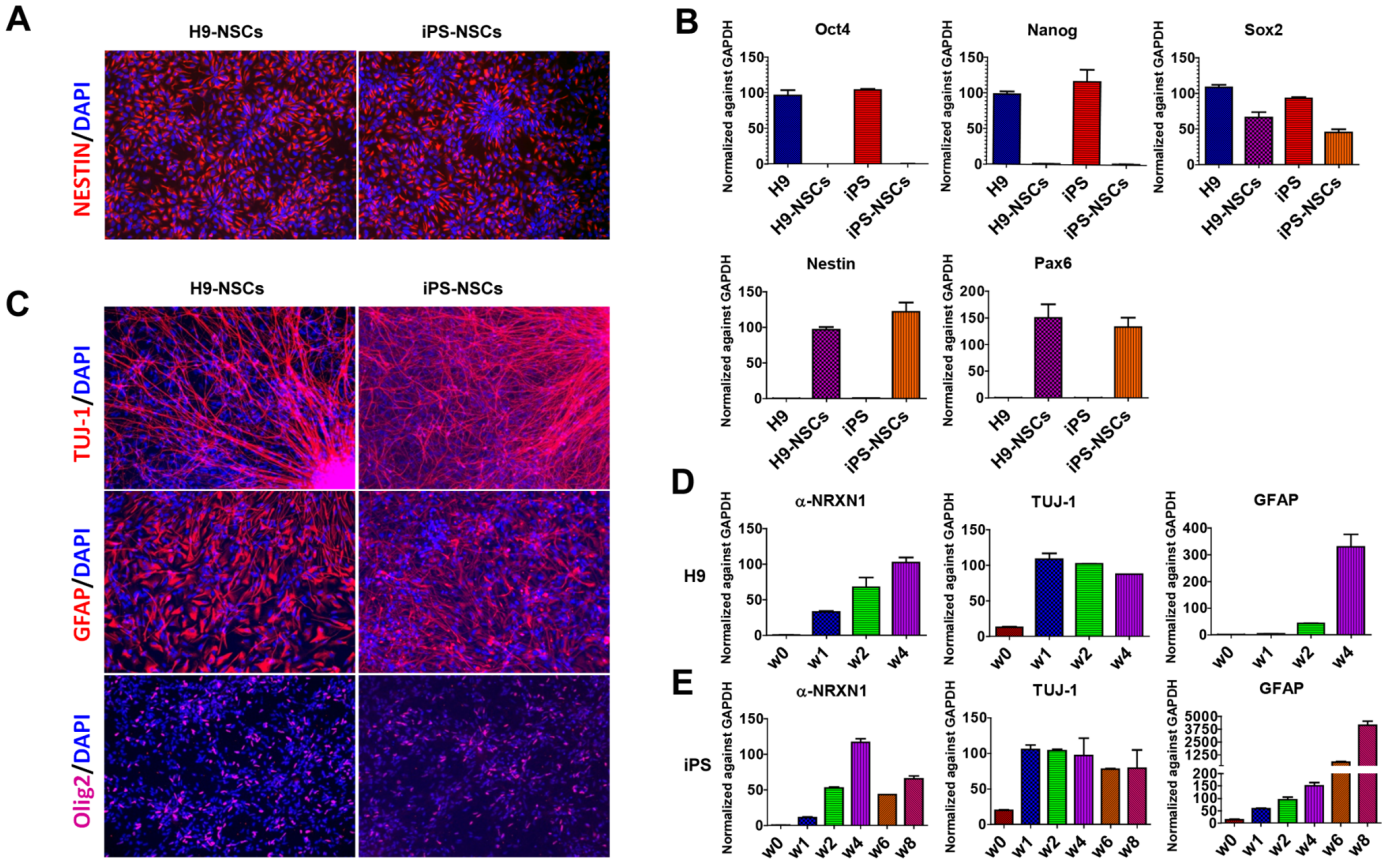
Neural stem cells (NSCs) derived from human embryonic stem cells H9 and hiPS maintain differentiation potential. **A.** NESTIN staining indicates that close to 100% positive NSCs are derived from H9 and hiPS. **B.** qPCR showed that hESCs (H9) and iPS highly express pluripotency markers *Oct4, Nanog* and *Sox2*, yet NSCs highly express NSCs markers *Pax6* and *Nestin*. **C.** H9 and hiPS derived NSCs can differentiate into both neural and glial lineage as stained by neuron marker *TUJ-1*, astrocyte marker *GFAP* and oligodendrocyte marker *Olig2*. **D.** H9 and **E.** iPS derived NSCs differentiated in time-dependent manner, with predicated gene expression pattern. *w*, abbr. of week.

We obtain neural stem cells (NSCs) by differentiating hiPSCs into embryoid bodies followed by neurospheres formation, from which we obtained monolayer neural rosettes and neural progenitors. In parallel, we also examined the ES cell line H9 using the same set of experimental procedures, to compare the results with those obtained from hiPSCs. We subsequently characterized the feature of NSCs by immunocytochemistry (**Figure 2A**), and found that almost all the cells were Nestin positive. Additionally, qPCR analysis showed that the NSCs-specific markers *NESTIN* and *PAX6* are highly expressed in NSCs, yet *OCT4* and *NANOG* cannot be detected (**Figure 2B**), suggesting that no pluripotent stem cells remained. Results from the hiPSCs and hESCs are highly consistent.

We next examined the differentiation potential of NSCs, as well as the expression patterns of neuronal marker TUJ-1, astrocyte marker GFAP and oligodendrocyte marker Olig2 in NSCs. Growth factor bFGF was removed to permit NSCs differentiation for 14 days. The differentiated cells were fixed and immunocytochemistry was performed. Meanwhile, cells in parallel plates were collected at multiple time points for gene expression analysis. As shown in **Figure 2C**, NSCs derived from hESCs (H9) and hiPSCs possessed the differentiation potential to generate 3 lineages of central nerve system. qPCR data revealed that after removal of growth factors, the expression of neuron marker *TUJ-1* was immediately induced to the highest level in one week in control cell line treated with non-targeting shRNAmir, suggesting the immediate differentiation of neurons after the initiation of induction. After that, *TUJ-1* expression slowly decreased but remained at high level for several weeks (**Figure 2D, E**). *NRXN1* expression was gradually induced and reached the peak around 4 weeks post differentiation (**Figure 2D, E**). The expression of astrocyte marker GFAP was very low in the early differentiation stage, but gradually increased in a time-dependent manner and finally reached peak at week 8 in our observation window for the hiPSCs (**Figure 2E**). Being consistent with the known physiological developmental pattern and neurogenesis process that NSCs generate neurons before generating glia cells [57], our results suggested that NSCs derived from hESCs and hiPSCs also have the same differentiation potential and pattern. We noted that in a recent time-course analysis of differentiated primary normal human neuronal progenitors (NHNP), the authors also found that neural specification may be entrained at time point week 4 [31]. These data demonstrated that NSCs derived from stem cells represent features of endogenous NSCs to a large extent, and that they could be used as cellular models for investigating neurodevelopment *in vitro*.

### Reduction of *NRXN1* Alters Neuronal Pathways during Neurodevelopment

We next validated a shRNAmir-based inducible system for knocking down α-*NRXN1* gene expression, by testing the system in HEK-293T cells first. The cells have endogenous α-*NRXN1* and β-*NRXN1* expression as shown by qPCR and Western blot (**Figure S1 in File S1**). We transfected the shRNAmir into HEK-293T cells and used doxycycline to induce expression of shRNAmir. As indicated by qPCR and Western blot (**Figure S2 in File S1**), α-*NRXN1* gene expression was reduced significantly after doxycycline induction for 5 days, suggesting the effectiveness of this system. We evaluated three clones of the shRNAmir designed for *NRXN1* (sh1, sh2, sh3), and selected sh2 and sh3 for follow-up experiments due to their ability to knockdown *NRXN1* efficiently and consistently in H9-derived NSCs (**Figure S3 in File S1**). For comparison, a non-targeting shRNAmir with the similar vector design was used as control.

**Figure 3:**
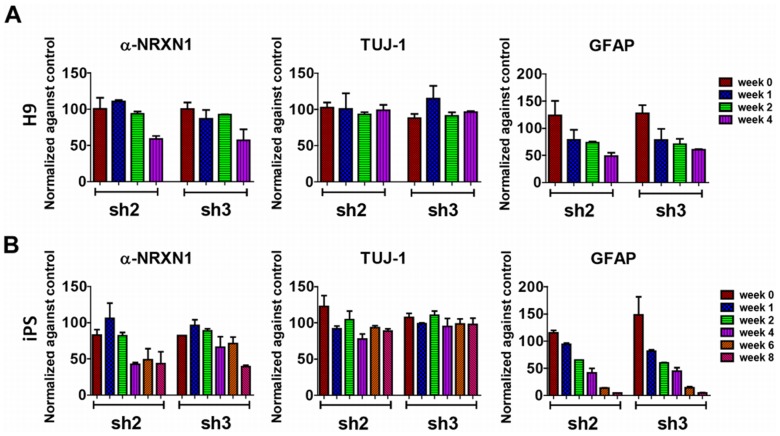
α-*NRXN1* knockdown block astrocytes differentiation in time-dependent manner. **A.** and **B.** shRNAmir knockdown of α-*NRXN1* in H9 (A) and iPS (B) have >50% knockdown efficiency in a time-dependent manner, and block the astrocytes differentiation in a time-dependent manner, without influencing neuronal differentiation. *sh2*: shRNAmir clone V2THS_68983; *sh3*: shRNAmir clone V2THS_246996.

Following this, we packaged shRNAmir into lentivirus particles and infected hiPSCs-derived NSCs and hESCs-derived NSCs. To estimate the knockdown efficiency of *NRXN1* in NSCs, doxycycline was added into NSCs cultures at the beginning of the differentiation stage to induce shRNA expression. We observed strong Red Fluorescence Protein (RFP) signals from the NSCs, suggesting that shRNAmir was successfully induced and expressed in all NSCs studied (**Figure S4 in File S1**). As a quick validation, qPCR and Western blot were performed on NSCs 5 days post doxycycline induction, and the results confirmed similar knockdown effect in NSCs as in HEK-293T cells (**Figure S3 in File S1**). During a few weeks post induction, RNA was isolated at selected time points and qPCR analysis showed that *NRXN1* expression was effectively reduced by ∼50% around week 4 for both hiPSCs and hESCs in comparison to controls with non-targeting shRNAmir (**Figure 3A, B**). Possibly due to the fact that α-*NRXN1* was upregulated immediately after the initial differentiation process and reached the peaks around 4 weeks post differentiation (**Figure 2D,E**), the efficiency of knockdown reached the highest level around 4 weeks, and maintained up to 8 weeks (**Figure 2E**). To examine the transcriptome-level changes as a result of *NRXN1* perturbation, we used next-generation sequencing (RNA-Seq) to examine the transcriptome in the NSCs derived from hiPSCs with and without *NRXN1* knockdown, at week 0 and week 4. The Illumina HiSeq2000 sequencer was used to generate 91 bp paired end sequencing data; on average, over 60 million reads were obtained for each cell line. RNA-Seq data showed that α-*NRXN1* level was reduced by ∼45% at week 4 after knockdown, which was consistent with the qPCR data, demonstrating the effectiveness and accuracy of RNA-Seq. The other two major β-*NRXN1* isoforms level were reduced ∼30–50% at the same time by RNA-Seq, suggesting the global effect of shRNAmir knockdown on multiple *NRXN1* isoforms (**Figure S5 in File S1**).

To examine the biological pathways and genetic networks that were affected by *NRXN1* knockdown, we applied two complementary analytical approaches. First, we identified 138 genes that are differentially expressed at week 4 between NSCs with or without *NRXN1* knockdown (**Table S1 in File S1**). A pathway enrichment analysis using DAVID web server [52] identified multiple Gene Ontology (GO) categories that were significantly altered (**Table 1**), including cell adhesion (20 genes, P = 2.8×10^−6^, FDR = 3.2×10^−3^) and neuron differentiation (13 genes, P = 2.1×10^−4^, FDR = 7.6×10^−2^). *NRXN1* is the only overlapping gene in these two pathways. Given that *NRXN1* functions in cell-cell adhesion and neurodevelopment, the results from the functional enrichment analysis suggested that perturbations of *NRXN1* can impact the whole functional pathway that *NRXN1* is involved in. We also found that two transcription-related pathways in the “Molecular Function” GO domain were differentially expressed, and they contain largely overlapping genes. We annotated the statistical significance for differential expression and the known functionality for all the genes in cell adhesion, neuron differentiation and transcription factor activity pathways (**Table S2 in File S1**). Some of the genes, such as *NCAN* and *EPHA7*, have similar functionality as *NRXN1* in mediating neuronal cell interactions, while some other genes, such as *PCDH19*, *CNTN3*, *CTNNA3*, *LMX1B*, are known autism candidate genes [58].

**Table 1:** Biological pathways that are differentially expressed between neurons with or without *NRXN1* knockdown at week 4.

Name (Category)^1^	Count	P-value	FDR^2^	Genes
cell adhesion (GOTERM_BP_FAT)	20	2.8×10–6	3.2×10–3	CD47, F11R, SOX9, CTNNA3, CXCL12, CLDN6, COL4A6, CNTN3, FLRT2, FBLN5, ITGA7, MGP, MMRN1, NRXN1, NCAN, NPTN, PCDH19, TNC, THBS1, TGFBI
sequence-specific DNA binding (GOTERM_MF_FAT)	17	8.7×10–6	2.1×10–3	ELF4, FOSL2, LHX1, LHX5, LMX1B, NKX6-1, SOX9, DLX5, EGR1, ONECUT2, OTP, PRRX1, PHOX2B, SHOX2, TSHZ3, ZEB1, ZFHX3
neuron differentiation(GOTERM_BP_FAT)	13	2.1×10–4	7.6×10–2	EPHA7, LHX1, LHX5, LMX1B, NKX6-1, CXCL12, DLX5, NRXN1, NUMB, ONECUT2, OTP, PAX2, SLC1A3
transcription factor activity (GOTERM_MF_FAT)	19	2.5×10–4	2.9×10–2	ELF4, FOSL2, LHX1, LHX5, LMX1B, NKX6-1, SOX9, DLX5, EGR1, NFAT5, ONECUT2, OTP, PRRX1, PHOX2B, SHOX2, TSHZ3, TRIM22, ZEB1, ZFHX3

1 Enrichment for these functional categories are computed by the DAVID web server [52] on Gene Ontology (GO) Biological Process or Molecular Function categories with > = 10 genes. The GO FAT categories are a subset of filtered GO terms. One pathway (“biological adhesion”) was omitted from the table as it is identical to “cell adhesion”.

2 FDR was calculated by Benjamini-Hochberg adjustment [80].

**Figure 4:**
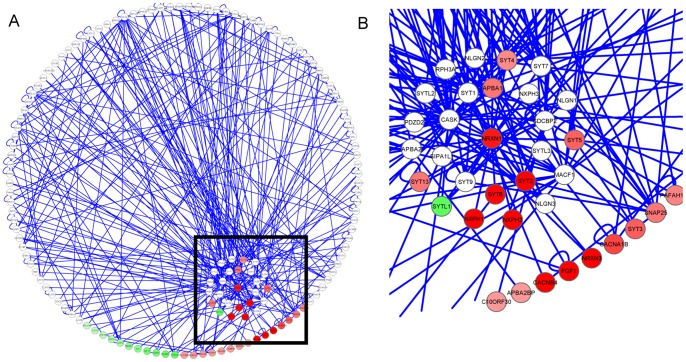
A network of known protein-protein interactions that includes all first-degree neighbors (direct interaction partners) and second-degree neighbors (interaction partners with first-degree neighbors) of *NRXN1*. (A) First degree neighbors are plotted surrounding *NRXN1*, while second-degree neighbors are plotted in the outer circle. Genes with differential expression P-values less than 0.05 are colored by their fold change values (red: down-regulated, green: up-regulated), with higher color intensity indicating higher fold changes. B, a zoomed-in view of the portion of the network surrounding *NRXN1* (black square in panel A). Multiple genes that directly interact with *NRXN1* are down-regulated as a result of *NRXN1* knockdown.

Next, we analyzed molecular networks affected by *NRXN1* knockdown using relaxed cutoff values (P<0.05) for differential expression. Based on known protein-protein interactions [53], [54], we constructed a *NRXN1*-centric molecular network using all genes that were connected to *NRXN1* by at most two degrees of separation (**Figure 4**). This interactome dataset was generated by merging molecular interaction data from a variety of sources and was provided by Cytoscape (http://wiki.cytoscape.org/Data_Sets). We colored genes with differential expression in red (down-regulation) or green (up-regulation), with the intensity of the color corresponding to the expression fold changes. It is clear that a large fraction (32%) of the *NRXN1* interactors tend to have decreased expression levels as a result of *NRXN1* knockdown. These include several synaptotagmins (SYT2, SYT4, SYT5, SYT6, SYT13), which are integral membrane proteins of synaptic vesicles and mediate calcium-dependent regulation of membrane trafficking in synaptic transmission [59], [60]. These also include several neurexophilins (NXPH1, NXPH2), which are neuropeptide-like glycoproteins that form a very tight complex with neurexins [61] and APBA1, a putative vesicular trafficking protein that couples synaptic vesicle exocytosis to neuronal cell adhesion [62]. In comparison, only a small fraction (15%) of second-degree neighbors of *NRXN1* (genes connected to *NRXN1* interactors) had decreased expression levels. For all human genes, this fraction dropped down to 4%, suggesting that neighbors of *NRXN1* in the molecular interaction network were more likely to be down-regulated by *NRXN1* knockdown (P = 3×10^−12^ by two-sided Fisher’s exact test for first-degree interactors). We recognize that gene function and interaction annotations for human genome are not yet comprehensive or accurate enough; nevertheless, these complementary analyses further confirmed that single-gene perturbation can influence molecular networks that are connected or related to the target gene.

Additionally, we also performed differential expression analysis between week 0 and week 4 for NSCs with *NRXN1* knockdown and without knockdown, respectively. This analysis helps identify genes that have altered expression levels during neurodevelopment, and helps examine whether *NRXN1* knockdown affects these genes. With the RNA-Seq data, we identified 511 differentially expressed genes between week 0 and week 4 in NSCs with knockdown, and 566 genes in NSCs without knockdown, including 377 overlapping genes. Among the 179 genes that do not reach statistical significance for differential expression between week 0 and 4 as a result of *NRXN1* knockdown, we noted several neurexins (*NRXN1*, *NRXN2*) and glutamate receptors (*GRIA2*, *GRIA4*, *GRIK3*). Functional enrichment analysis showed that this list is highly enriched for genes involved in nervous system development (P = 1×10^−10^, FDR = 6.9×10^−8^), suggesting that *NRXN1* knockdown affects the time-course expression regulation of these genes.

### *NRXN1* Knockdown may Affect Astrocytes Differentiation during Neurodevelopment

**Figure 5:**
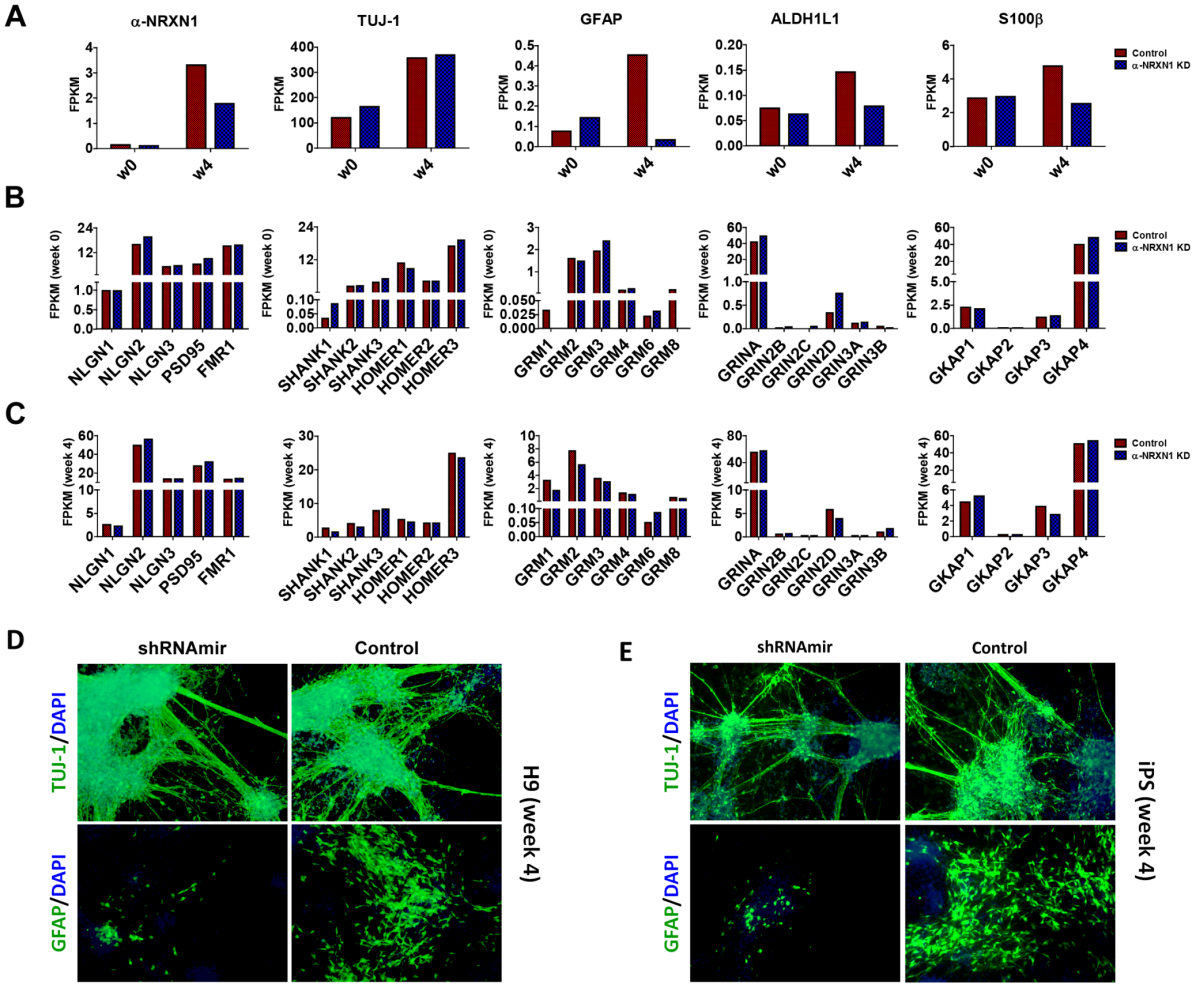
RNA-Seq analysis indicated that astrocytes differentiation is blocked by α-*NRXN1* knockdown in iPS cells, and it is not mediated by post-synaptic pathway associated with *NRXN1*. **A.** α-*NRXN1* knockdown blocked astrocytes differentiation rather than neuronal differentiation in week 4 of iPS compared to week 0. Neuron marker: *TUJ-1*; Astrocyte markers: *GFAP, ALDH1L1* and *S100β*. **B.** and **C.** post-synaptic pathway associated with *NRXN1* is not influenced by α-NRXN1 knockdown. *(B)* week 0; *(C)* week 4. **D.** and **E.** immunocytochemical staining of *TUJ-1* and *GFAP* indicate that astrocytes differentiation is blocked at week 4 in H9 (D) and iPS (E) in shRNAmir (sh2) expressing cells. *NLGN*, Neuroligin; *FMR1*, fragile X mental retardation protein; *GRIN*, N-methyl-D-aspartate receptors (NMDARs); *GRM*, metabotropic glutamate receptors (mGluRs), *GKAP*, guanylate kinase-associated proteins. *w*, abbr. of week.

We did not observe obvious morphological differences between cells with or without *NRXN1* knockdown, and we investigated whether the differentiation potential and patterns differ between these cells. As previously shown in qPCR (**Figure 3**), TUJ-1 remained largely unchanged, suggesting that cells with *NRXN1* knockdown still have intact neuronal differentiation potential; however, we are interested in the developmental time course and the fate commitments to specific lineages. Large discordance between controls and *NRXN1* depleted NSCs was observed on GFAP expression, which is a marker for the astrocytes lineage. As GFAP started to increase dramatically after week 4 in the control cells with non-targeting shRNAmir (**Figure 2 D,E**), the relative reduction of GFAP gene expression in cells with *NRXN1* knockdown compared to controls followed a clear time-dependent pattern (**Figure 3**). Similarly, based on RNA-Seq data (**Figure 5A**), comparing cells with *NRXN1* knockdown to those without knockdown by non-targeting shRNAmir, we observed decreased expression for several astrocyte markers including GFAP (∼93%), ALDH1L1 [57], [63] (∼46%) and S100β [64] (∼47%), yet GAPDH expression remained almost identical. In comparison, many molecules involved in post-synaptic complex, such as NLGNs, PSD95, GKAPs, FMR1, mGLURs (GRMs), SHANKs, HOMERs and NMDARs (GRINs), had little changes (**Figure 5B,C**). Therefore, apart from the direct outcome of *NRXN1* deficiency on gene expression of neuronal pathways, the indirect effects on astrocytes might also be intriguing, considering the role of astrocytes in synapse formation, maturation, efficacy, and plasticity [57], [65]. However, we recognize that there is no known evidence that neurexin-neuroligin connections play a role in astrocytes development. We also note that the developmental time course of neurons and astrocytes in our study closely match known development process in human brain [57]: indeed, comparison to previously published gene expression data across multiple time points on the developing human brains (spanning from embryonic development to late adulthood) suggests that week 4 in NSCs development corresponds to ∼13–16 post-conceptional weeks in the developing human brains [66] (**Figure S6 in File S1**).

To further investigate how *NRXN1* knockdown affects astrocytes development and differentiation, we also carried out immunocytochemistry experiments for parallel plates that were used in qPCR for H9 cells (**Figure 5D**) and iPS cells (**Figure 5E**). Clearly, staining of TUJ-1 and GFAP indicated that astrocytes differentiation was significantly blocked on week 4 in hESCs and hiPSCs that were expressing shRNAmir against *NRXN1*. Additionally, we also observed that the general trend of gene expression for *NRXN1* correlated with *GFAP* well in control cells and in cells with *NRXN1* knockdown; for example, for sh2-treated NSCs derived from hiPSCs, the correlation coefficient across an 8-week time period was 0.84 (**Figure 3B**). Our observations suggested that *NRXN1* knockdown might lead to the inhibition of astrocytes differentiation, due to different fate commitment of NSCs to different lineages. However, we recognize an alternative explanation: *NRXN1* knockdown may merely delay normal time course of neuronal differentiation, and the reduced astrocytes markers reflect slower neuronal development, given that gliogenesis generally followed neurogenesis during differentiation of NSCs. Finally, we wish to point out that although *GFAP* is commonly used as an astrocytes marker, several reports also demarcate an important role for this molecule in the neurogenesis and neuronal proliferation [67]. Therefore, additional follow-up studies are necessary to confirm if *NRXN1* loss of function leads to inhibition of astrocytes differentiation.

## Discussion

In the current study, we investigated the functional significance of *NRXN1* deletions in neurodevelopment, using hiPSCs and hESCs as *in vitro* models. During differentiation of neural stem cells into mature neurons, we found that knockdown of a single neuronal gene *NRXN1* leads to systematic perturbations of expression levels of several neurodevelopment-related pathways. Additionally, we also observed reduced astrocytes differentiation potential, and a strong positive correlation between expression of α-*NRXN1* and the astrocyte marker *GFAP*. Altogether, our results suggested that *NRXN1* deletion most likely influenced the synapse function and neuronal connectivity. The relationship between *NRXN1* knockdown and inhibition of astrocytes differentiation is intriguing and is worthy of follow-up studies. Both the disturbed neuronal pathways and the inhibition of astrocytes differentiation could play a role in the molecular pathophysiology of several neurodevelopmental diseases, such as autism and schizophrenia.

One of the interesting findings in our study is the reduced astrocytes generation from neural stem cells after *NRXN1* knockdown. Astrocytes are star shaped glial cells present in central nerve system and spinal cord, and they have several important functions [68], [69]. They are the most abundant cell types in mammalian brains and are involved in the physical structure and buffer of the neural system. They could regulate the electrical pulse between neurons, control the neural transmitters uptake and release, modulate synapse transmission and help repair the neural system. Astrocytes could also form synapse between neural synapse and release gliotransmitter, forming tripartite synapse and dynamically regulate the synapse transmission [65]. The delicate tree structure of astrocytic cell and the fact that each astrocyte can potentially contact 300,000 neurons, provide the topology that ensures multiple levels of interaction between neurons and astrocytes. Although astrocytes are not capable to generate electrical signals like neurons, they respond with elevated intracellular calcium concentration to mechanical or chemical stimulus [70]. As the astrocytes actively participate in neuron synapse formation and signing transduction, the defect of astrocytes function may have severe consequence for neural system. Previous studies already suggested that the abnormal glial-neuronal communication may be involved in pathogenesis of autism [71], [72] and schizophrenia [73]. Given the strong association between astrocytes and the synapse, it is reasonable to speculate that the deletion of *NRXN1* may influence astrocytes differentiation, thus contributing to pathogenesis of diseases with impaired synaptic adhesion and transmission.

Recent advances in hESCs and hiPSCs research have made it possible to establish *in vitro* model systems to study complex neurodevelopmental disorders, for which animal models are generally not available or not ideal to use in specific scientific contexts. Neuronal system is generally difficult to study, as live neurons are not readily available from the patients to understand the molecular pathophysiology of the diseases. Although hESCs are more widely used for studies of molecular mechanisms, we expect that patient-specific hiPSCs will find more use in future genetic studies. These iPS cells can be generated from skin fibroblasts or peripheral blood monocytes [74], which share the same genetic background as the patients themselves, thus enabling more refined studies *in vitro*. By analyzing morphological, electrophysiological, transcriptional and functional differences of neurons derived from specific patients and control subjects, we may better understand the molecular mechanism of diseases pathogenesis, and ultimately help develop better individualized diagnosis and therapeutic tools.

We believe that one of the previously unrecognized advantages of using stem cells model to study complex neuropsychiatric diseases is to understand the functional impacts of CNVs by modulating gene expression. CNVs may account for a significant proportion of human phenotypic variation, including disease susceptibility [75]–[77]. Deletion or duplications of one or more genes may lead to dosage-dependent gene expression changes, disrupt regulatory elements, generate novel fusion products, or act by way of position effect, with various possible positive and negative consequences, including imprinting and differential allelic expression [78], [79]. Given that several single-gene deletions and recurrent genomic deletions serve as highly penetrant disease susceptibility factors in neuropsychiatric diseases, it is expected that stem cell models can be readily used to screen the functional significance of CNVs in large scale, using a combination of gene knockin and knockdown techniques, coupled with cell biology studies (morphological analysis, electrophysiology analysis, etc) and molecular biology studies (gene expression, methylation, histone modification, etc).

In conclusion, the combination of stem cells models with targeted gene knockdown and high-throughput transcriptome sequencing clearly provided a novel approach for studying the functional significance of CNVs in complex neuropsychiatric diseases. The models we established here would help confirm the roles of candidate CNVs or other genetic alternations identified from previous genetic studies (CNV association, candidate gene association and genome-wide association studies), and help discover additional diseases susceptibility genes and pathways. Finally, these *in vitro* models may facilitate drug discovery by serving as therapeutic models for drug screening, and may facilitate pharmacogenomic studies to understand how differences in genetic background impact treatment responses.

## Supporting Information

File S1 Figure S1. Quantitative real-time PCR (a) and Western blot (b) show that HEK-293T cells have native *NRXN1* expression including α-*NRXN1* and two short β-*NRXN1* isoforms. S1, S2, S3, S4: Four HEK-293T cells from 4 different frozen stocks. Figure S2. Evaluation of the knockdown efficiency of shRNAmir clones against *NRXN1* using HEK-293T cells. All three shRNAmir clones have significant knockdown of α-*NRXN1* shown by quantitative real-time PCR (A) and Western blot (B). *sh1*: shRNAmir clone V2THS_246980; *sh2*: shRNAmir clone V2THS_68983; *sh3*: shRNAmir clone V2THS_246996. Each shRNAmir clone has 4 biological replicates for knockdown experiments in the HEK-293T cells, labeled as 1,2,3,4. *w*, abbr. of week. Figure S3. Quantitative real-time PCR (a) and Western blot (b) show that 5 days after Doxycycline induction in H9-derived NSCs, three shRNAmir have different but significant knockdown efficiency. *sh1*: shRNAmir clone V2THS_246980; *sh2*: shRNAmir clone V2THS_68983; *sh3*: shRNAmir clone V2THS_246996. Figure S4. Doxycycline induced highly expressed RFP reporter gene inside neurons (A) and astrocytes-like cells (B) in NSCs derived from H9 or iPS at week 2, week 3 and week 4 post differentiation. Figure S5. RNA-Seq data indicated that the shRNAmir (V2THS_68983) also has significant knockdown efficiency to two major β-*NRXN1* isoforms on RefSeq ID NM_004801.4 (A) and NM_138735.2 (B) on week 4 of hiPSCs. *w*, abbr. of week. Figure S6. Dynamic gene expression along entire development and adulthood in the cerebellar cortex (CBC), mediodorsal nucleus of the thalamus (MD), striatum (STR), amygdala (AMY), hippocampus (HIP) and 11 areas of neocortex (NCX), in the developing human brains. Week 4 in our study appears to correspond to period 4 (13–16 post-conceptional weeks) in the Human Brain Transcriptome data generated by Kang et al (2011). Table S1. A list of genes with significantly altered gene expression levels at week 4 as a result of *NRXN1* knockdown by shRNAmir (FDR<0.01), compared to the cells with non-targeting shRNAmir. Table S2. Known functions for the list of genes in cell adhesion, neuron differentiation and transcription factor activity pathways shown in Table 1, based on the UniProt Knowledge Base.
(DOC)
