# Supplementary material for: Functional Impacts of NRXN1 Knockdown on Neurodevelopment in Stem Cell Models
Source: PLoS One. 2013 Mar 25;8(3):e59685. doi: 10.1371/journal.pone.0059685 (PMC3607566; doi:10.1371/journal.pone.0059685)
Supplement: File S1 — Figure S1. Quantitative real-time PCR (a) and Western blot (b) show that HEK-293T cells have native NRXN1 expression including α-NRXN1 and two short β-NRXN1 isoforms. S1, S2, S3, S4: Four HEK-293T cells from 4 different frozen stocks. Figure S2. Evaluation of the knockdown efficiency of shRNAmir clones against NRXN1 using HEK-293T cells. All three shRNAmir clones have significant knockdown of α-NRXN1 shown by quantitative real-time PCR (A) and Western blot (B). sh1: shRNAmir clone V2THS_246980; sh2: shRNAmir clone V2THS_68983; sh3: shRNAmir clone V2THS_246996. Each shRNAmir clone has 4 biological replicates for knockdown experiments in the HEK-293T cells, labeled as 1,2,3,4. w, abbr. of week. Figure S3. Quantitative real-time PCR (a) and Western blot (b) show that 5 days after Doxycycline induction in H9-derived NSCs, three shRNAmir have different but significant knockdown efficiency. sh1: shRNAmir clone V2THS_246980; sh2: shRNAmir clone V2THS_68983; sh3: shRNAmir clone V2THS_246996. Figure S4. Doxycycline induced highly expressed RFP reporter gene inside neurons (A) and astrocytes-like cells (B) in NSCs derived from H9 or iPS at week 2, week 3 and week 4 post differentiation. Figure S5. RNA-Seq data indicated that the shRNAmir (V2THS_68983) also has significant knockdown efficiency to two major β-NRXN1 isoforms on RefSeq ID NM_004801.4 (A) and NM_138735.2 (B) on week 4 of hiPSCs. w, abbr. of week. Figure S6. Dynamic gene expression along entire development and adulthood in the cerebellar cortex (CBC), mediodorsal nucleus of the thalamus (MD), striatum (STR), amygdala (AMY), hippocampus (HIP) and 11 areas of neocortex (NCX), in the developing human brains. Week 4 in our study appears to correspond to period 4 (13–16 post-conceptional weeks) in the Human Brain Transcriptome data generated by Kang et al (2011). Table S1. A list of genes with significantly altered gene expression levels at week 4 as a result of NRXN1 knockdown by shRNAmir (FDR<0.01), compared to the ce [file pone.0059685.s001.doc]

**Supplementary Figures and Tables**

**Figure S1.** Quantitative real-time PCR (a) and Western blot (b) show that HEK-293T cells have native *NRXN1* expression including α-*NRXN1* and two short β-*NRXN1* isoforms. S1, S2, S3, S4: Four HEK-293T cells from 4 different frozen stocks.


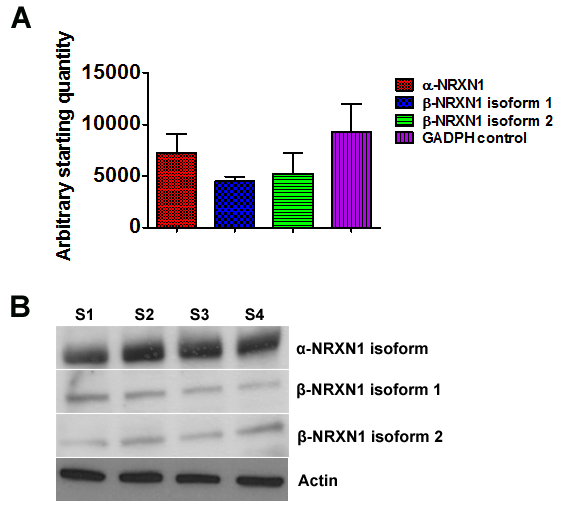


**Figure S2.** Evaluation of the knockdown efficiency of shRNAmir clones against *NRXN1* using HEK-293T cells. All three shRNAmir clones have significant knockdown of α-*NRXN1* shown by quantitative real-time PCR (A) and Western blot (B). *sh1*: shRNAmir clone V2THS_246980; *sh2*: shRNAmir clone V2THS_68983; *sh3*: shRNAmir clone V2THS_246996. Each shRNAmir clone has 4 biological replicates for knockdown experiments in the HEK-293T cells, labeled as 1,2,3,4. *w*, abbr. of week.

**
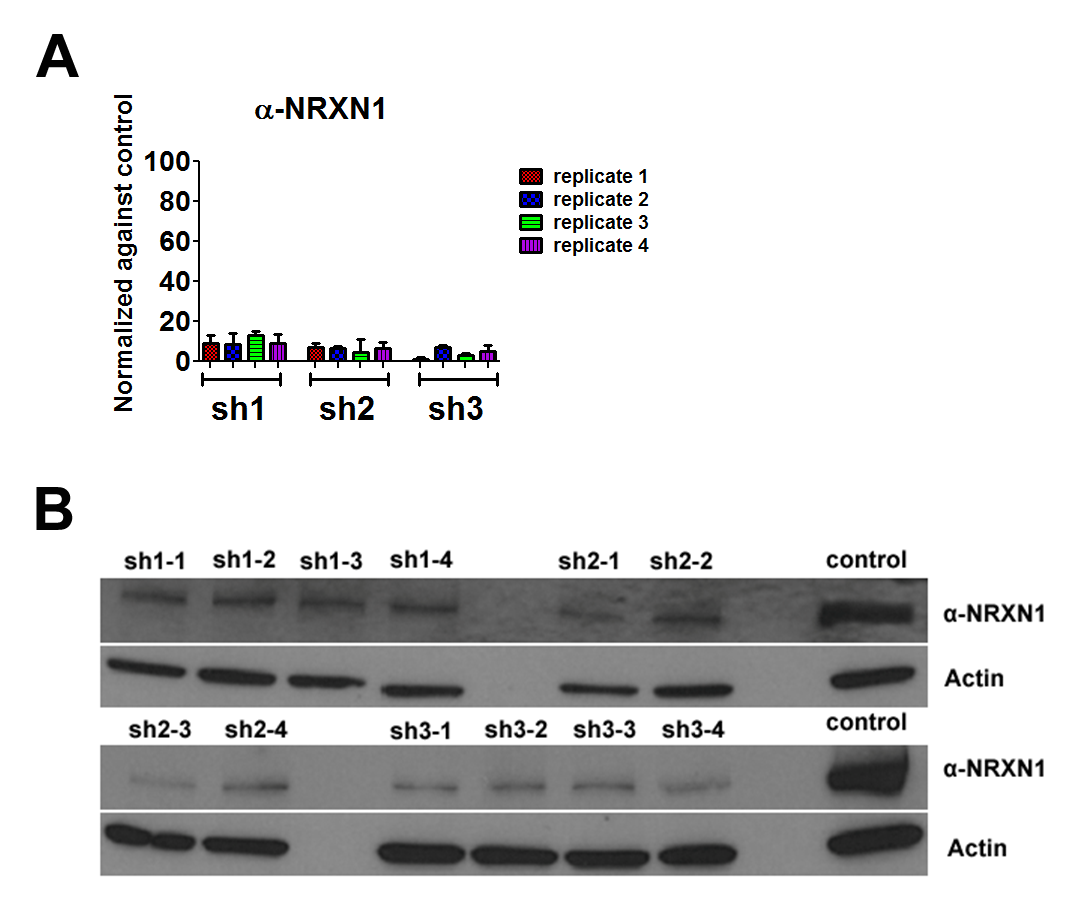
**

**Figure S3.** Quantitative real-time PCR (a) and Western blot (b) show that 5 days after Doxycycline induction in H9-derived NSCs, three shRNAmir have different but significant knockdown efficiency. *sh1*: shRNAmir clone V2THS_246980; *sh2*: shRNAmir clone V2THS_68983; *sh3*: shRNAmir clone V2THS_246996.


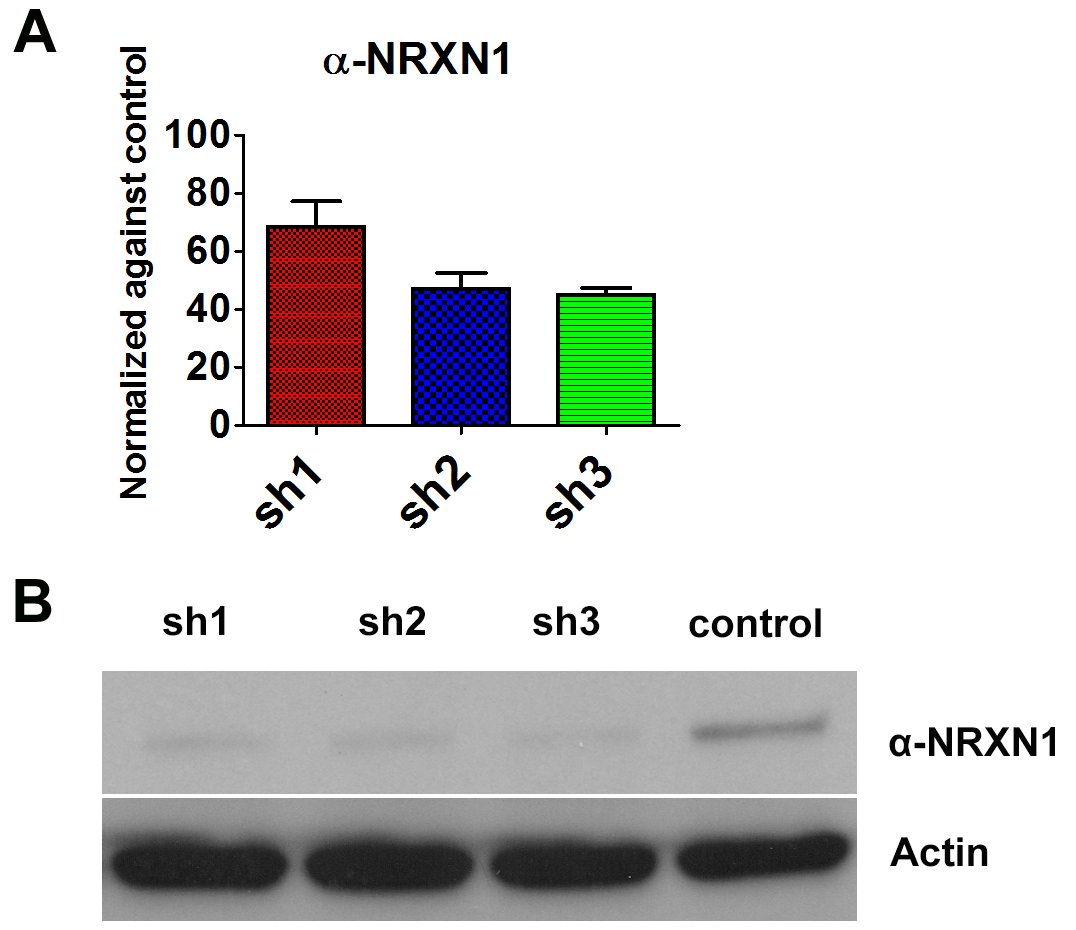


**Figure S4:** Doxycycline induced highly expressed RFP reporter gene inside neurons (**A**) and astrocytes-like cells (**B**) in NSCs derived from H9 or iPS at week 2, week 3 and week 4 post differentiation.


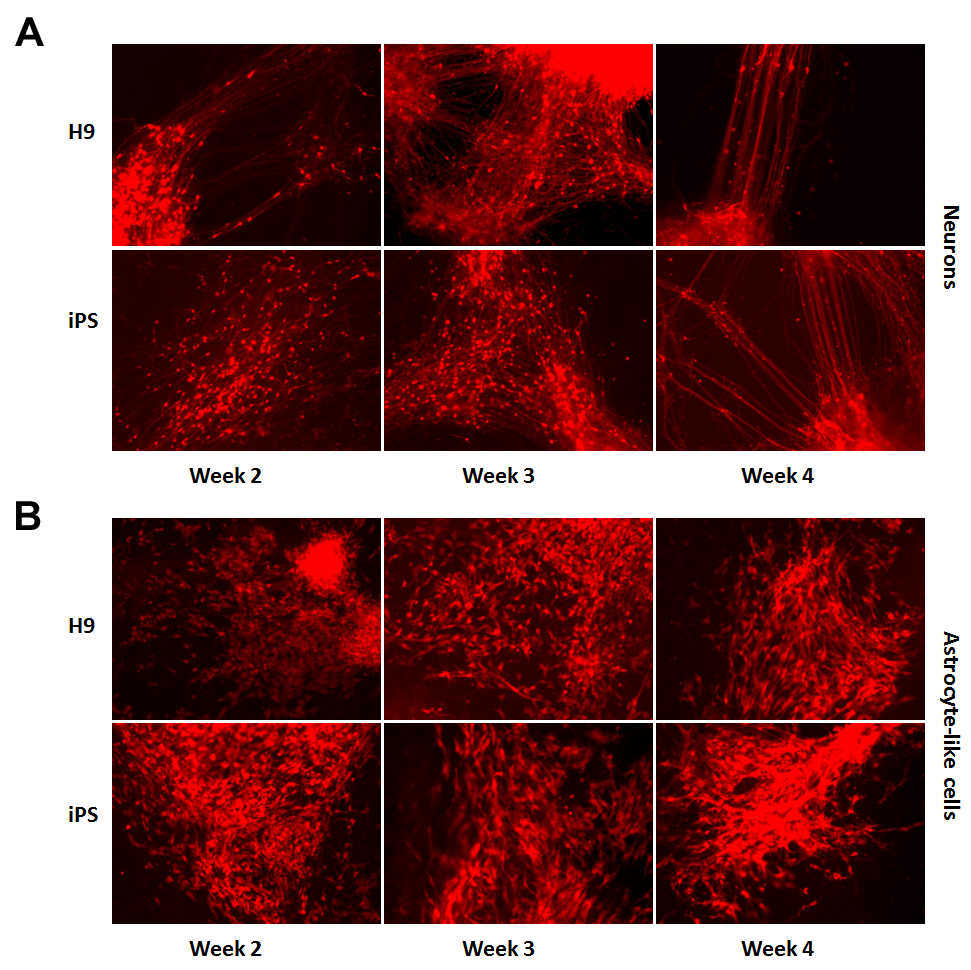


**Figure S5:** RNA-Seq data indicated that the shRNAmir (V2THS_68983) also has significant knockdown efficiency to two major β-*NRXN1* isoforms on RefSeq ID NM_004801.4 (**A**) and NM_138735.2 (**B**) on week 4 of hiPSCs. *w*, abbr. of week.


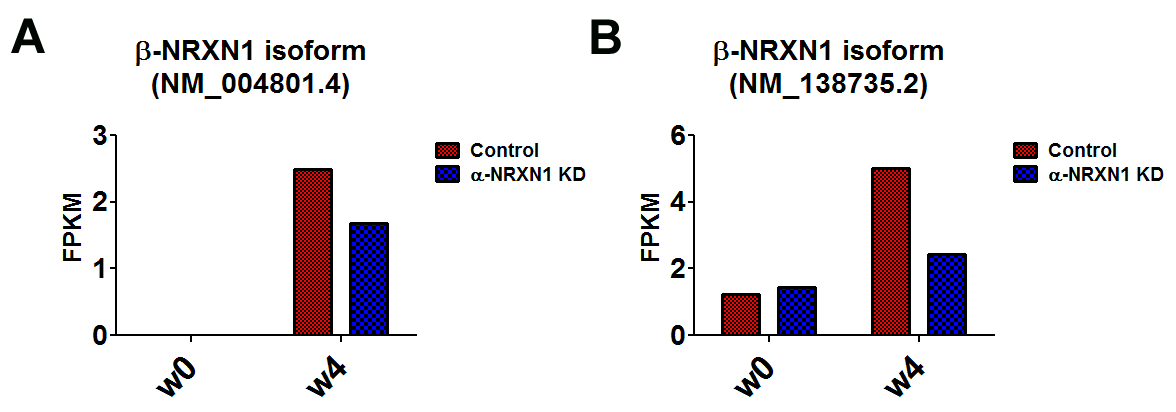


**Figure S6:** Dynamic gene expression along entire development and adulthood in the cerebellar cortex (CBC), mediodorsal nucleus of the thalamus (MD), striatum (STR), amygdala (AMY), hippocampus (HIP) and 11 areas of neocortex (NCX), in the developing human brains. Week 4 in our study appears to correspond to period 4 (13-16 post-conceptional weeks) in the Human Brain Transcriptome data generated by Kang et al (2011).


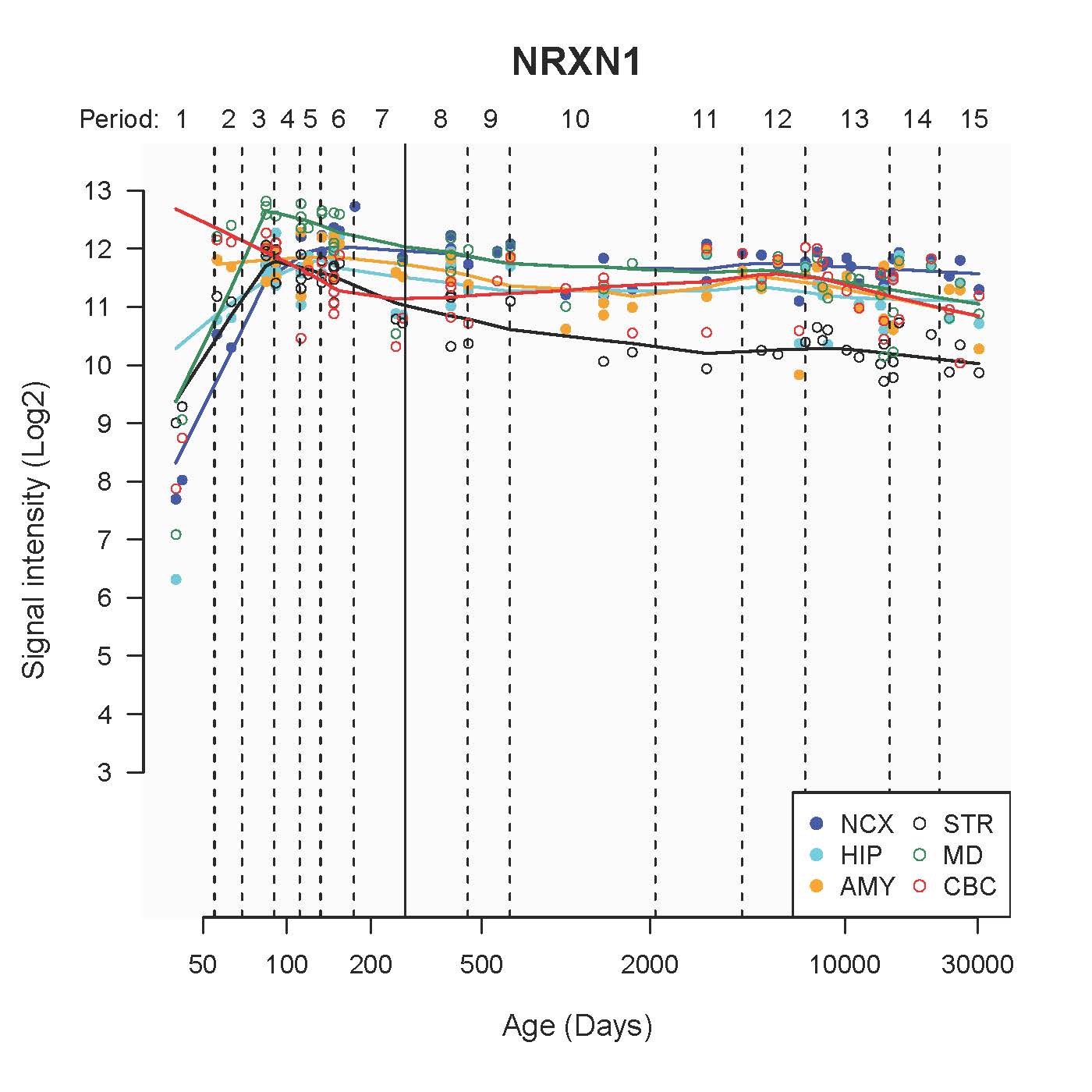

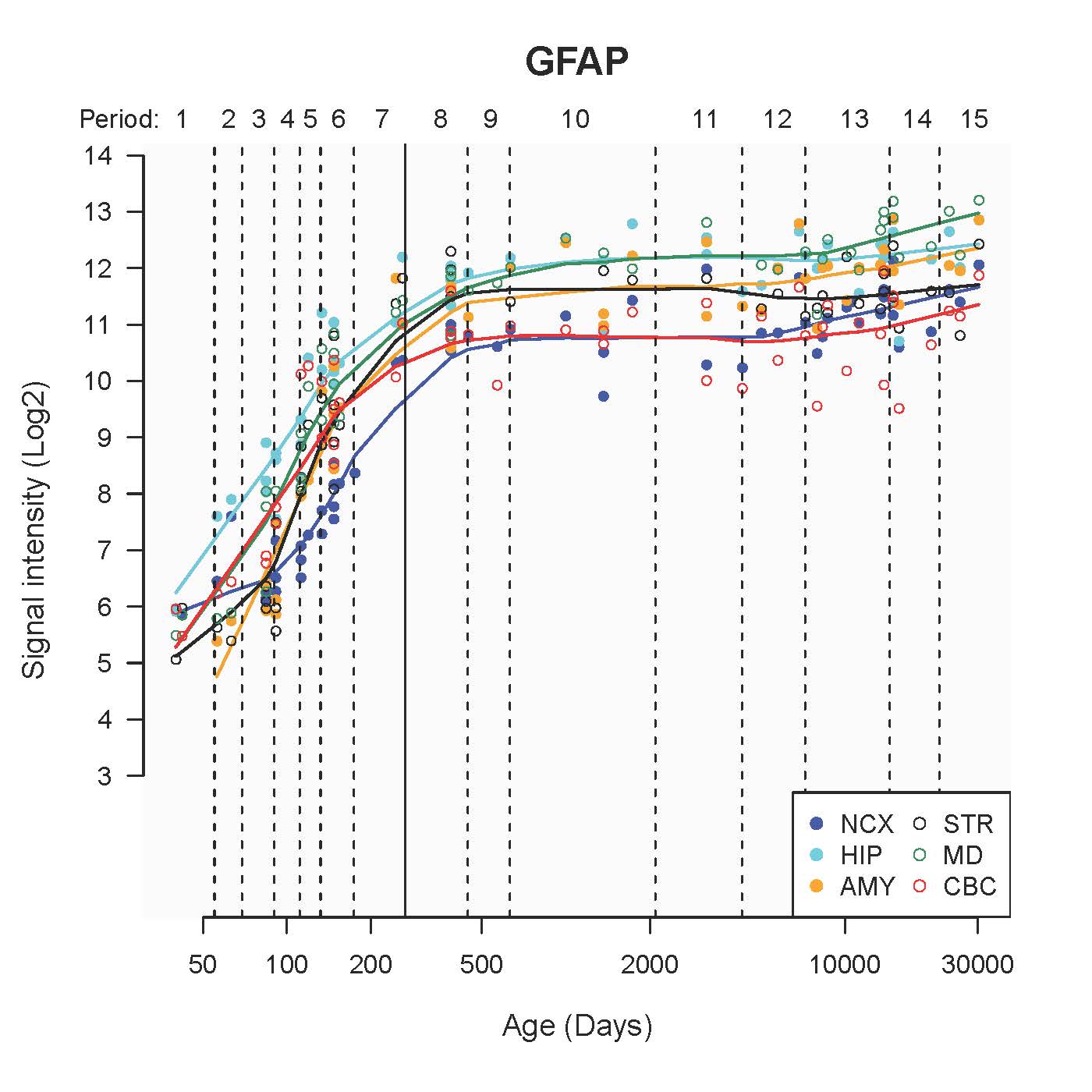


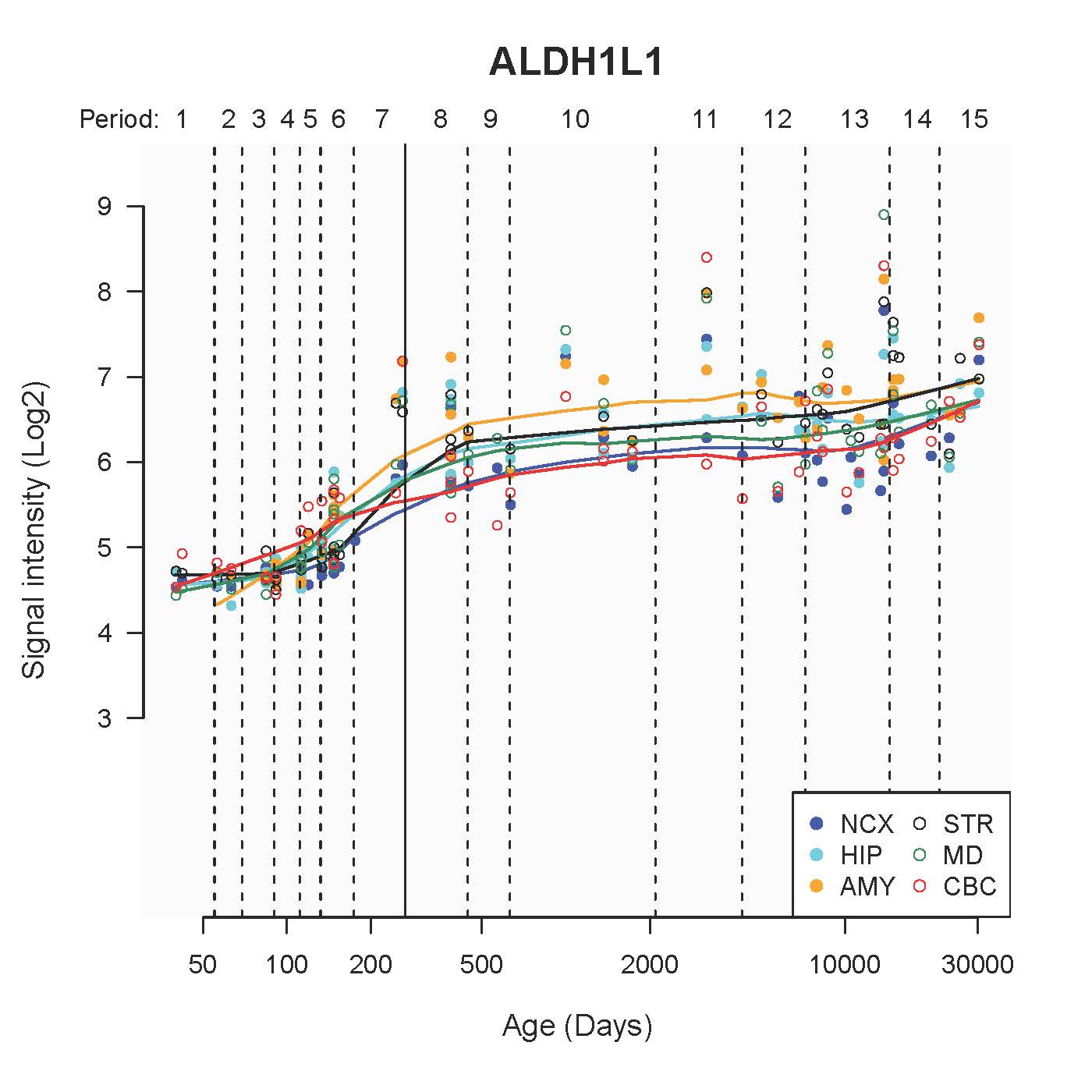

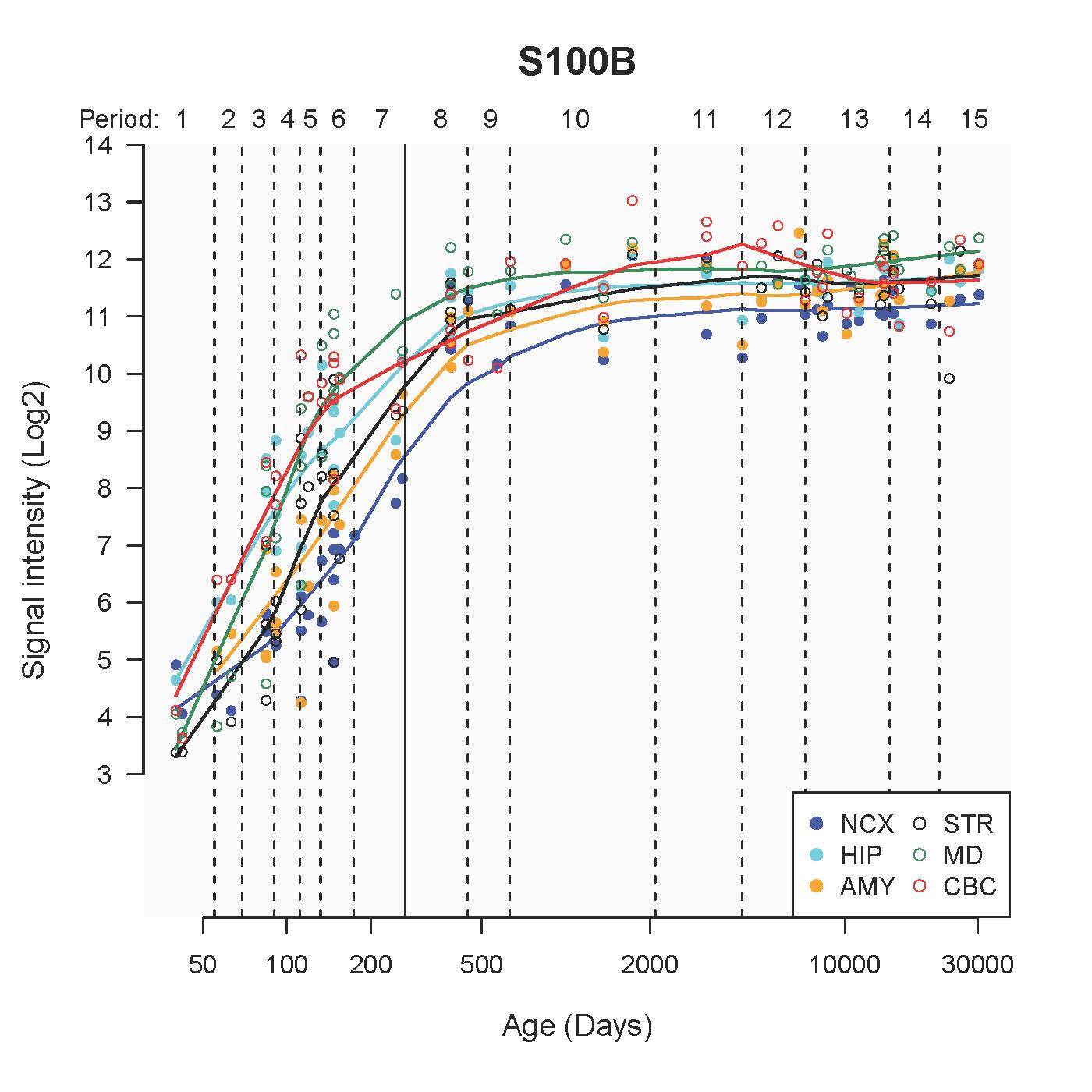


Table S1. A list of genes with significantly altered gene expression levels at week 4 as a result of *NRXN1* knockdown by shRNAmir (FDR<0.01), compared to the cells with non-targeting shRNAmir.

| **Gene** | **Locus** | **log2(fold_change)** | **P-value** | **FDR** |
| --- | --- | --- | --- | --- |
| ACTG2 | 2:74120092-74146780 | -0.966 | 0.000122937 | 0.00885827 |
| AHNAK | 11:62201015-62314332 | 0.699 | 1.50E-06 | 0.000224497 |
| ARHGEF4 | 2:131674223-131804836 | 0.713 | 2.03E-06 | 0.000291506 |
| ARL5A | 2:152657479-152685009 | NA | 4.49E-23 | 2.05E-20 |
| ATXN3 | 14:92524895-92572965 | NA | 4.97E-31 | 4.14E-28 |
| AXL | 19:41725107-41767671 | 0.816 | 7.13E-07 | 0.000124212 |
| BAMBI | 10:28966423-28971868 | 1.070 | 9.49E-06 | 0.00109896 |
| BMP5 | 6:55620235-55740375 | 0.750 | 3.93E-05 | 0.00374447 |
| BRUNOL4 | 18:34823002-35146000 | -0.719 | 2.26E-06 | 0.000318053 |
| BST2 | 19:17513754-17516384 | 1.839 | 1.57E-05 | 0.00174659 |
| C15orf40 | 15:83654994-83680393 | NA | 9.96E-17 | 3.80E-14 |
| C1orf61 | 1:156374054-156399184 | -2.284 | 3.85E-09 | 9.22E-07 |
| C20orf103 | 20:9495270-9511171 | -1.085 | 1.66E-07 | 3.17E-05 |
| C3orf32 | 3:8661316-8693737 | 1.069 | 2.91E-06 | 0.000390965 |
| CADPS | 3:62384020-62861064 | -0.799 | 6.07E-06 | 0.000730441 |
| CCPG1 | 15:55609381-55700574 | NA | 1.61E-29 | 1.23E-26 |
| CD47 | 3:107761940-107809935 | 0.478 | 9.27E-05 | 0.00722457 |
| CD70 | 19:6585849-6591163 | 1.063 | 3.53E-07 | 6.59E-05 |
| CLDN6 | 16:3064712-3068188 | 1.191 | 6.26E-06 | 0.000744256 |
| CNTN3 | 3:74311721-74570343 | 0.814 | 1.05E-06 | 0.000171853 |
| COL4A6 | X:107398836-107682704 | 0.790 | 7.77E-05 | 0.00635155 |
| CPA4 | 7:129932973-129964020 | 1.449 | 1.13E-06 | 0.000181618 |
| CRYAB | 11:111779349-111782473 | -1.888 | 0.000122589 | 0.00885827 |
| CTNNA3 | 10:67679724-69455949 | 1.161 | 4.61E-05 | 0.00409145 |
| CXCL12 | 10:44865604-44880545 | -1.183 | 3.10E-06 | 0.000405141 |
| D4S234E | 4:4387982-4543775 | -0.909 | 1.21E-08 | 2.70E-06 |
| DDB2 | 11:47236492-47260769 | 0.871 | 2.47E-06 | 0.000336851 |
| DIDO1 | 20:61509089-61569304 | NA | 8.19E-65 | 0 |
| DLX5 | 7:96649701-96654143 | -2.290 | 3.93E-09 | 9.22E-07 |
| DSP | 6:7540683-7586946 | 0.834 | 4.13E-06 | 0.000518865 |
| EGR1 | 5:137801180-137805004 | 1.583 | 4.44E-16 | 1.63E-13 |
| ELF4 | X:129198894-129244688 | 0.964 | 3.10E-06 | 0.000405141 |
| ELN | 7:73442426-73484237 | NA | 3.60E-28 | 2.53E-25 |
| EML2 | 19:46112657-46148775 | 0.875 | 3.24E-05 | 0.00329479 |
| EPHA7 | 6:93949737-94129300 | 0.607 | 0.000132703 | 0.00913058 |
| F11R | 1:160965000-160991133 | 0.749 | 3.44E-05 | 0.00335098 |
| FAM111B | 11:58874657-58894888 | NA | 8.75E-25 | 4.21E-22 |
| FAM98C | 19:38893774-38916945 | NA | 1.98E-11 | 5.83E-09 |
| FBLN5 | 14:92335754-92414046 | -1.774 | 2.18E-10 | 6.23E-08 |
| FER1L4 | 20:34146506-34195484 | 0.992 | 1.41E-06 | 0.000218921 |
| FLRT2 | 14:85996487-86094270 | -0.886 | 4.34E-05 | 0.00401109 |
| FOSL2 | 2:28607275-28637516 | 0.885 | 0.000128564 | 0.00894468 |
| FUS | 16:31191430-31206192 | -0.564 | 7.19E-07 | 0.000124212 |
| GFRA1 | 10:117816435-118033126 | -0.807 | 4.31E-07 | 7.89E-05 |
| GPBAR1 | 2:219125737-219128582 | 1.233 | 0.000126934 | 0.00894468 |
| GPR124 | 8:37654400-37707431 | -0.880 | 8.28E-05 | 0.00659189 |
| GPX8 | 5:54408798-54469005 | NA | 3.20E-26 | 1.83E-23 |
| GRIA4 | 11:105480799-105852819 | -0.897 | 2.93E-05 | 0.00301525 |
| HCN4 | 15:73612199-73661605 | -0.856 | 0.000135949 | 0.00921529 |
| HEATR1 | 1:236681564-236767841 | NA | 1.26E-31 | 1.16E-28 |
| HLA-E | 6:30457182-30461982 | 0.851 | 8.47E-06 | 0.000994168 |
| HSPA2 | 14:65007185-65009955 | 0.754 | 9.39E-05 | 0.00722457 |
| INPP5D | 2:234054794-234116549 | 0.982 | 8.33E-07 | 0.000141164 |
| ISLR2 | 15:74421714-74429143 | -1.174 | 3.00E-08 | 6.53E-06 |
| ITGA7 | 12:56075329-56106089 | 0.680 | 0.000105529 | 0.00791963 |
| KCNIP1 | 5:169780880-170163636 | NA | 2.22E-14 | 7.25E-12 |
| KIAA1161 | 9:34368906-34376894 | -1.029 | 0.000114729 | 0.00846679 |
| KLHDC9 | 1:161068150-161070138 | NA | 1.82E-13 | 5.55E-11 |
| KRT18 | 12:53342654-53346685 | 1.023 | 6.24E-05 | 0.00528992 |
| KRT8 | 12:53290970-53298868 | 0.946 | 1.37E-05 | 0.00154669 |
| LHX1 | 17:35294498-35300494 | -1.144 | 5.42E-05 | 0.00472192 |
| LHX5 | 12:113900693-113918286 | -1.326 | 2.12E-05 | 0.00223183 |
| LMX1B | 9:129376721-129463311 | -0.688 | 5.08E-06 | 0.000620251 |
| LOC100289627 | 5:180663927-180670914 | -0.935 | 2.24E-09 | 5.69E-07 |
| LOC100505584 | 1:33402049-33430286 | 2.234 | 0 | 0 |
| LOC100507207 | 8:24735953-24737342 | -1.544 | 8.46E-05 | 0.00667286 |
| LOC100507645 | 11:65236860-65273940 | 0.540 | 3.62E-06 | 0.000466899 |
| LOC254559 | 15:89921272-89941718 | -1.282 | 4.93E-07 | 8.85E-05 |
| LRRC17 | 7:102453307-102715288 | -1.051 | 2.07E-05 | 0.00220606 |
| LRRC8B | 1:89990396-90063420 | NA | 1.27E-31 | 1.16E-28 |
| LUM | 12:91497231-91505542 | -1.005 | 8.16E-05 | 0.00655388 |
| MAB21L1 | 13:35516423-36246873 | -0.807 | 4.91E-05 | 0.00431814 |
| MASP1 | 3:186933872-187009810 | -1.417 | 6.00E-15 | 2.11E-12 |
| MBNL1 | 3:151980404-152183569 | NA | 3.75E-32 | 4.29E-29 |
| MGP | 12:15034114-15038853 | -1.237 | 0.000120356 | 0.00881105 |
| MMRN1 | 4:90816051-90875780 | 0.652 | 0.000128997 | 0.00894468 |
| MT1E | 16:56659584-56661024 | 1.591 | 0.000101353 | 0.00772902 |
| MTMR11 | 1:149900542-149908791 | 0.818 | 3.32E-05 | 0.00333695 |
| MVP | 16:29827527-29859341 | 0.807 | 4.29E-05 | 0.00400387 |
| MYOF | 10:95066185-95242074 | 0.726 | 5.53E-05 | 0.00477462 |
| NCAN | 19:19322781-19363061 | -1.041 | 8.17E-10 | 2.27E-07 |
| NEBL | 10:21068901-21463116 | 0.643 | 2.45E-06 | 0.000336851 |
| NFAT5 | 16:69598996-69738553 | NA | 2.88E-46 | 4.40E-43 |
| NKX6-1 | 4:85414435-85419387 | -1.274 | 7.15E-05 | 0.00594494 |
| NPAS3 | 14:33408458-34273382 | -0.730 | 2.04E-06 | 0.000291506 |
| NPTN | 15:73735498-73925753 | NA | 1.17E-27 | 7.66E-25 |
| NRXN1 | 2:50145642-51259674 | -0.873 | 2.22E-09 | 5.69E-07 |
| NUMB | 14:73741917-73925286 | NA | 1.88E-32 | 2.46E-29 |
| ONECUT2 | 18:55102916-55158530 | -0.642 | 4.48E-05 | 0.00406105 |
| OTP | 5:76924536-76934522 | -2.427 | 0 | 0 |
| P4HA1 | 10:74766974-74856732 | 0.671 | 1.76E-06 | 0.000259151 |
| PAX2 | 10:102505467-102589698 | -2.137 | 6.35E-14 | 2.00E-11 |
| PCDH19 | X:99546641-99665271 | 0.515 | 6.66E-05 | 0.0055925 |
| PCYT1B | X:24576203-24690979 | 0.704 | 1.63E-07 | 3.17E-05 |
| PDGFB | 22:39619684-39640957 | 1.050 | 1.08E-05 | 0.0012392 |
| PHOX2B | 4:41746098-41750987 | -1.277 | 1.49E-06 | 0.000224497 |
| PLAU | 10:75669726-75682535 | 1.343 | 8.95E-07 | 0.000148963 |
| PLEKHA4 | 19:49340353-49371884 | 0.706 | 0.000129024 | 0.00894468 |
| PLXNA4 | 7:131808090-132333447 | -1.119 | 3.13E-09 | 7.75E-07 |
| PPL | 16:4932507-4987136 | 0.926 | 3.36E-05 | 0.00334046 |
| PRC1 | 15:91509267-91537804 | 0.487 | 0.000138679 | 0.00933123 |
| PRRX1 | 1:170633312-170708541 | -1.825 | 0 | 0 |
| PTRF | 17:40554466-40575338 | 0.732 | 0.000113123 | 0.00841614 |
| PTTG3P | 8:67624890-67774257 | NA | 9.36E-08 | 1.90E-05 |
| REEP1 | 2:86441115-86565206 | -0.990 | 1.25E-07 | 2.49E-05 |
| RELL1 | 4:37455551-37687999 | NA | 2.43E-17 | 9.68E-15 |
| RGS5 | 1:163112088-163172963 | 1.002 | 1.64E-05 | 0.00180987 |
| RHOC | 1:113243748-113250025 | 0.523 | 4.53E-05 | 0.00406527 |
| SEL1L3 | 4:25749048-25864610 | 0.817 | 4.14E-06 | 0.000518865 |
| SEMA3C | 7:80371853-80548667 | 0.779 | 1.77E-05 | 0.00192604 |
| SFPQ | 1:35649200-35658743 | -0.648 | 0.000124861 | 0.00892661 |
| SFRP1 | 8:41119475-41166990 | -1.083 | 1.19E-08 | 2.70E-06 |
| SHOX2 | 3:157813799-157823952 | -1.275 | 1.78E-09 | 4.78E-07 |
| SLC1A3 | 5:36606456-36688436 | -0.902 | 7.83E-08 | 1.63E-05 |
| SOX9 | 17:70117160-70122561 | -0.657 | 0.00013531 | 0.0092153 |
| SPARCL1 | 4:88394486-88450655 | 0.861 | 1.27E-06 | 0.000200179 |
| SUPT3H | 6:44796468-45518819 | NA | 3.17E-21 | 1.38E-18 |
| SUV39H2 | 10:14920781-14946314 | NA | 2.14E-27 | 1.31E-24 |
| TGFBI | 5:135364583-135399507 | -0.982 | 3.39E-05 | 0.00334046 |
| THBS1 | 15:39873279-39889668 | 0.704 | 5.05E-06 | 0.000620251 |
| TMEM151B | 6:44238479-44247182 | -0.820 | 3.48E-05 | 0.00335098 |
| TMOD2 | 15:52043757-52108560 | -0.726 | 5.93E-05 | 0.00507054 |
| TNC | 9:117782804-117880486 | -0.956 | 7.15E-08 | 1.52E-05 |
| TNFSF9 | 19:6531009-6535939 | 1.136 | 2.03E-05 | 0.00219044 |
| TP53RK | 20:45186461-45318276 | NA | 7.42E-25 | 3.77E-22 |
| TRIL | 7:28992973-28998029 | -0.950 | 7.77E-05 | 0.00635155 |
| TRIM22 | 11:5710918-5732093 | 0.729 | 4.12E-05 | 0.00388468 |
| TRIP10 | 19:6739706-6751529 | 0.758 | 0.000149826 | 0.00993517 |
| TSHZ3 | 19:31765850-31840190 | -0.823 | 0.000105583 | 0.00791963 |
| UACA | 15:70946892-71055850 | 0.606 | 9.37E-05 | 0.00722457 |
| VAT1L | 16:77822482-78014001 | 0.663 | 2.80E-05 | 0.00291508 |
| WASF2 | 1:27732125-27816669 | 0.628 | 8.13E-05 | 0.00655388 |
| WDR20 | 14:102606211-102690010 | NA | 1.32E-25 | 7.09E-23 |
| WRAP53 | 17:7571719-7606820 | NA | 1.08E-19 | 4.48E-17 |
| XRCC4 | 5:82373316-82649579 | NA | 9.28E-15 | 3.15E-12 |
| ZEB1 | 10:31608100-31818742 | NA | 1.01E-63 | 1.85E-60 |
| ZFHX3 | 16:72816783-73092534 | -0.495 | 4.48E-05 | 0.00406105 |
| ZFP36 | 19:39897486-39900045 | 1.053 | 0.000146388 | 0.00977809 |

Table S2. Known functions for the list of genes in cell adhesion, neuron differentiation and transcription factor activity pathways shown in Table 1, based on the UniProt Knowledge Base.

| **Gene** | **P-value** | **FDR** | **expression** | **Function (UniProtKB)** |
| --- | --- | --- | --- | --- |
| CD47 | 9.27E-05 | 0.00722457 | up | Has a role in both cell adhesion by acting as an adhesion receptor for THBS1 on platelets, and in the modulation of integrins. Plays an important role in memory formation and synaptic plasticity in the hippocampus |
| CLDN6 | 6.26E-06 | 0.000744256 | up | Plays a major role in tight junction-specific obliteration of the intercellular space |
| CNTN3 | 1.05E-06 | 0.000171853 | up | Contactins mediate cell surface interactions during nervous system development. |
| COL4A6 | 7.77E-05 | 0.00635155 | up | Major structural component of glomerular basement membranes (GBM), forming a 'chicken-wire' meshwork together with laminins, proteoglycans and entactin/nidogen |
| CTNNA3 | 4.61E-05 | 0.00409145 | up | Involved in formation of stretch-resistant cell-cell adhesion complexes |
| CXCL12 | 3.10E-06 | 0.000405141 | down | Activates the C-X-C chemokine receptor CXCR4 to induce a rapid and transient rise in the level of intracellular calcium ions and chemotaxis |
| F11R | 3.44E-05 | 0.00335098 | up | Plays a role in epithelial tight junction formation. |
| FBLN5 | 2.18E-10 | 6.23E-08 | down | Promotes adhesion of endothelial cells through interaction of integrins and the RGD motif. |
| FLRT2 | 4.34E-05 | 0.00401109 | down | May have a function in cell adhesion and/or receptor signaling. |
| ITGA7 | 0.000105529 | 0.00791963 | up | It is involved in the maintenance of the myofibers cytoarchitecture as well as for their anchorage, viability and functional integrity. |
| MGP | 0.000120356 | 0.00881105 | down | Associates with the organic matrix of bone and cartilage. |
| MMRN1 | 0.000128997 | 0.00894468 | up | Carrier protein for platelet (but not plasma) factor V/Va. Plays a role in the storage and stabilization of factor V in platelets. |
| NCAN | 8.17E-10 | 2.27E-07 | down | May modulate neuronal adhesion and neurite growth during development by binding to neural cell adhesion molecules |
| NPTN | 1.17E-27 | 7.66E-25 | up | Involved in long term potentiation at hippocampal excitatory synapses through activation of p38MAPK. May also regulate neurite outgrowth by activating the FGFR1 signaling pathway |
| NRXN1 | 2.22E-09 | 5.69E-07 | down | Function in the vertebrate nervous system as cell adhesion molecules and receptors |
| PCDH19 | 6.66E-05 | 0.0055925 | up | Potential calcium-dependent cell-adhesion protein |
| SOX9 | 0.00013531 | 0.0092153 | down | Plays an important role in the normal skeletal development. May regulate the expression of other genes involved in chondrogenesis by acting as a transcription factor for these genes |
| TGFBI | 3.39E-05 | 0.00334046 | down | Plays an important role in cell-collagen interactions. |
| THBS1 | 5.05E-06 | 0.000620251 | up | Adhesive glycoprotein that mediates cell-to-cell and cell-to-matrix interactions. |
| TNC | 7.15E-08 | 1.52E-05 | down | Implicated in guidance of migrating neurons as well as axons during development, synaptic plasticity, and neuronal regeneration |
| CXCL12 | 3.10E-06 | 0.000405141 | down | Activates the C-X-C chemokine receptor CXCR4 to induce a rapid and transient rise in the level of intracellular calcium ions and chemotaxis |
| DLX5 | 3.93E-09 | 9.22E-07 | down | Transcriptional factor involved in bone development. |
| EPHA7 | 0.000132703 | 0.00913058 | up | Receptor tyrosine kinase which binds promiscuously GPI-anchored ephrin-A family ligands residing on adjacent cells, leading to contact-dependent bidirectional signaling into neighboring cells. |
| LHX1 | 5.42E-05 | 0.00472192 | down | May play a role in early mesoderm formation and later in lateral mesoderm differentiation and neurogenesis. |
| LHX5 | 2.12E-05 | 0.00223183 | down | Plays an essential role in the regulation of neuronal differentiation and migration during development of the central nervous system |
| LMX1B | 5.08E-06 | 0.000620251 | down | Essential for the specification of dorsal limb fate at both the zeugopodal and autopodal levels. |
| NKX6-1 | 7.15E-05 | 0.00594494 | down | Binds to the insulin promoter and is involved in regulation of the insulin gene. Together with NKX2-2 and IRX3 acts to restrict the generation of motor neurons to the appropriate region of the neural tube. |
| NRXN1 | 2.22E-09 | 5.69E-07 | down | Function in the vertebrate nervous system as cell adhesion molecules and receptors |
| NUMB | 1.88E-32 | 2.46E-29 | down | Required throughout embryonic neurogenesis to maintain neural progenitor cells, also called radial glial cells (RGCs), by allowing their daughter cells to choose progenitor over neuronal cell fate |
| ONECUT2 | 4.48E-05 | 0.00406105 | down | Transcriptional activator. Activates the transcription of a number of liver genes such as HNF3B |
| OTP | 0 | 0 | down | Probably involved in the differentiation of hypothalamic neuroendocrine cells |
| PAX2 | 6.35E-14 | 2.00E-11 | down | Has a critical role in the development of the urogenital tract, the eyes, and the CNS. |
| SLC1A3 | 7.83E-08 | 1.63E-05 | down | Essential for terminating the postsynaptic action of glutamate by rapidly removing released glutamate from the synaptic cleft. |
| DLX5 | 3.93E-09 | 9.22E-07 | down | Transcriptional factor involved in bone development. |
| EGR1 | 4.44E-16 | 1.63E-13 | up | Activates the transcription of target genes whose products are required for mitogenesis and differentiation. |
| ELF4 | 3.10E-06 | 0.000405141 | up | Transcriptional activator. Plays a role in the development and function of NK and NK T-cells and in innate immunity. |
| FOSL2 | 0.000128564 | 0.00894468 | up | Controls osteoclast survival and size. activates LIF transcription, Activates CEBPB transcription |
| LHX1 | 5.42E-05 | 0.00472192 | down | May play a role in early mesoderm formation and later in lateral mesoderm differentiation and neurogenesis. |
| LHX5 | 2.12E-05 | 0.00223183 | down | Plays an essential role in the regulation of neuronal differentiation and migration during development of the central nervous system |
| LMX1B | 5.08E-06 | 0.000620251 | down | Essential for the specification of dorsal limb fate at both the zeugopodal and autopodal levels. |
| NFAT5 | 2.88E-46 | 4.40E-43 | up | Plays a role in the inducible expression of genes. Regulates hypertonicity-induced cellular accumulation of osmolytes |
| NKX6-1 | 7.15E-05 | 0.00594494 | down | Binds to the insulin promoter and is involved in regulation of the insulin gene. Together with NKX2-2 and IRX3 acts to restrict the generation of motor neurons to the appropriate region of the neural tube. |
| ONECUT2 | 4.48E-05 | 0.00406105 | down | Transcriptional activator. Activates the transcription of a number of liver genes such as HNF3B |
| OTP | 0 | 0 | down | Probably involved in the differentiation of hypothalamic neuroendocrine cells |
| PHOX2B | 1.49E-06 | 0.000224497 | down | Involved in the development of several major noradrenergic neuron populations, including the locus coeruleus. |
| PRRX1 | 0 | 0 | down | Acts as a transcriptional regulator of muscle creatine kinase (MCK) and so has a role in the establishment of diverse mesodermal muscle types |
| SHOX2 | 1.78E-09 | 4.78E-07 | down | May be a growth regulator and have a role in specifying neural systems involved in processing somatosensory information, as well as in face and body structure formation. |
| SOX9 | 0.00013531 | 0.0092153 | down | Plays an important role in the normal skeletal development. May regulate the expression of other genes involved in chondrogenesis by acting as a transcription factor for these genes |
| TRIM22 | 4.12E-05 | 0.00388468 | up | Interferon-induced antiviral protein involved in cell innate immunity. |
| TSHZ3 | 0.000105583 | 0.00791963 | down | Transcriptional regulator involved in developmental processes. Regulates the development of neurons involved in both respiratory rhythm and airflow control. Promotes maintenance of nucleus ambiguus (nA) motoneurons |
| ZEB1 | 1.01E-63 | 1.85E-60 | down | Sequence-specific DNA binding transcription factor activity. Binding Zinc ion. |
| ZFHX3 | 4.48E-05 | 0.00406105 | down | Binding nucleic acid and zinc ion. |
